# Translating dietary standards into healthy meals with few-ingredient substitutions

**DOI:** 10.1371/journal.pdig.0001367

**Published:** 2026-05-28

**Authors:** Trevor Chan, Ilias Tagkopoulos

**Affiliations:** 1 Department of Computer Science, University of California, Davis, California, United States of America; 2 Genome Center, University of California, Davis, California, United States of America; 3 USDA/NSF AI Institute for Next Generation Food Systems (AIFS), University of California, Davis, California, United States of America; The University of Sheffield, UNITED KINGDOM OF GREAT BRITAIN AND NORTHERN IRELAND

## Abstract

An important goal for personalized diet systems is to improve nutritional quality without compromising convenience or affordability. We present an end-to-end framework that converts dietary standards into complete meals with few ingredient changes. Using the What We Eat in America (WWEIA) intake data for 135,491 meals, we identify 34 interpretable meal archetypes that we then use to condition a generative model and a portion predictor to meet USDA nutritional targets. In comparisons within archetypes, generated meals show a median 47.0% reduction in absolute deviation from per-meal RDI targets, while remaining compositionally close to real meals. Our results show that by allowing one to three food substitutions, we were able to create meals that were 10% more nutritious, while reducing costs 19–32%, on average. By turning dietary guidelines into realistic, budget-aware meals and simple swaps, this framework can support public-health programs and consumer apps; clinical decision support is a promising future direction pending further validation and safety review, to deliver scalable, equitable improvements in everyday nutrition.

## Author summary

Healthy eating advice often asks people to change too much at once. We developed a framework that starts from meals people already eat and then makes small, practical changes to improve nutritional quality. Using U.S. national dietary intake data, we identified common meal patterns for breakfast, lunch, and dinner, then trained a generative model to create realistic meals within those patterns. A separate portioning step adjusted serving sizes so meals better matched USDA dietary targets. Compared with observed meals in the same meal pattern, generated meals showed substantially better alignment with nutrient recommendations while staying compositionally similar to real meals. We then tested one-, two-, and three-item substitutions to see whether meals could be improved with minimal disruption. Across feasible substitutions, these small changes often improved nutritional quality while also lowering modeled meal cost. This work suggests that dietary guidelines can be translated into realistic, affordable meal options without requiring complete changes in eating habits. With further validation, this kind of framework could support public health nutrition programs and consumer-facing diet tools.

## Introduction

Diet is one of the most powerful, modifiable drivers of obesity, diabetes, cardiovascular disease, and other non-communicable conditions, yet translating nutrition science into day-to-day meals remains difficult for most people [1]. Personalized diet recommendation systems promise scale and individualization, but many tools still optimize a single goal (taste, calories, or convenience), lack strict standards-based evaluation, and provide limited guidance on how to change as little as possible to eat better [2]. Consequently, there remains a gap between guideline-concordant diets and what recommenders reliably generate in practice [2]. Rule-based and expert-curated systems helped encode guidelines but often sacrificed adaptability and user fit [3]. Subsequent machine-learning approaches - clinical optimization for chronic disease (e.g., DietOS) and IoMT-assisted personalization - improved targeting, yet frequently treated health metrics in isolation and rarely reported controlled benchmarking against USDA nutrient standards [3,4]. Many-objective/evolutionary methods began balancing adequacy, preferences, and diversity, and clustering/classification pipelines introduced segmentation, but most do not close the loop from what to eat (composition) to how much to eat (portions), a tradeoff which is a key determinant of adequacy, balance, and moderation in real meals [5,6] and knowledge graph, health-aware recommenders [7]. Generative modeling has accelerated progress towards this goal. Systems, such as Yum-Me, explicitly model both nutrient goals and taste [8], while recent pipelines leveraging methods like variational autoencoders produce plausible meal plans [9]. More prevalent LLM-based approaches also explore more interactive suggestions [10]. However, systematic reviews highlight inconsistency and factual errors in LLM-generated nutrition, underscoring the need for domain constraints and transparent, multi-objective evaluation [11–14]. For real-world impact in nutrition science and clinical informatics, tools must embed dietary standards, quantify uncertainty, and deliver actionable and few ingredient changes that users can implement without overhauling habits [15,16].

**Fig 1:**
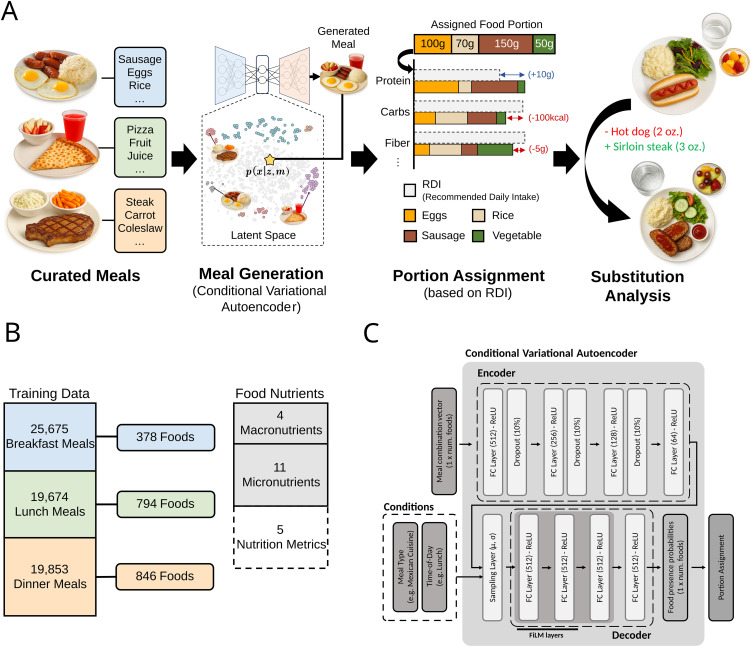
End-to-end meal generation, RDI-aware portioning, and substitution evaluation. **(A)** We start from curated meals and train a conditional variational autoencoder (CVAE) that samples realistic food combinations for a chosen archetype (e.g., breakfast) from a structured latent space. A portion assigner initializes standard servings and then adjusts grams to meet USDA RDI/AMDR targets while preserving the combination. Downstream, we evaluate fewest food-change substitutions by constrained nearest-neighbor search over real meals to find swaps that improve nutrition at lower or comparable cost under a portion-based restaurant pricing model. **(B)** Data and nutrients used for training and evaluation. Counts of meals per time-of-day, the number of foods available for each meal type, and the nutrient panel (4 macronutrients, 11 micronutrients, and 5 diet quality metrics). **(C)** The encoder/decoder of the CVAE are conditioned on archetype cluster and meal type via Feature-wise Linear Modulation (FiLM) layers. The decoder outputs food-presence probabilities which feed the portion assignment module. Meal images shown in this figure were generated by the authors using ChatGPT/OpenAI and are not reproduced or adapted from third-party published or copyrighted sources.

Here, we present an end-to-end framework for meal generation, and substitution (Fig 1A) designed for public-health impact. We use a training set of 65,202 meals, consisting of 2,018 foods (1,475 of them unique), each associated with 20 nutrients and other nutrient-metrics (Fig 1B), and we partition them into 34 archetypes (e.g., protein and grains) based on food categories and nutritional content. Then we train a Conditional Variational Autoencoder (CVAE), conditioned to these archetypes to generate representative meals that are subsequently compared to real meals (Fig 1C).

## Methods

### Data description

The USDA “What We Eat in America” (WWEIA) component of the National Health and Nutrition Examination Survey (NHANES) was the primary source for all analyses [17–20]. We used six survey waves (2013–2020), comprising 55,228 respondents and 135,491 meals (Table A in S1 File). WWEIA provides a hierarchical taxonomy of ingredients, foods, and meals indexed by USDA codes. Foods are constructed from ingredients and meals are constructed from foods. We standardized food codes across survey waves using USDA’s discontinuation and renumbering documentation and retained dropped or revised codes where mapping was unambiguous. The final corpus contained 8,650 food codes and 2,940 ingredient codes. We excluded pre-2013 surveys because discontinuation mappings were incomplete, preventing reliable code harmonization. Definitions for terms used throughout are given in Table B in S1 File. The pseudocode for the complete framework, which includes the data preprocessing, meal clustering, conditional meal generation, RDI portion assignment, and substitution optimization is provided in the Section D in S1 File.

### Data processing

The WWEIA dataset underwent comprehensive preprocessing to ensure data quality and reduce dimensionality. For surveys with discontinued food code documentation, we created a comprehensive dataset based on the discontinuation code given (Table C in S1 File). For expanded, consolidated, and renumbered food codes, all previous food codes affected were replaced with the most recent food codes. Dropped and revised food codes were kept in the dataset. This yielded a dataset with 120,375 meals and 6,212 foods. Of these meals, 39,749 were breakfast meals, 37,397 were lunch meals, and 43,229 were dinner meals. Data was organized into time-of-day meal-to-food data subsets with corresponding gram amounts. These datasets were further reduced to binary data that represented the presence or absence of food per time-of-day meal. Local Outlier Factorization (LOF) [21] was applied to meals: we used a contamination rate of 0.3%, removing the most extreme composition outliers based on the negative outlier factorization score, which resulted in the removal of 120 breakfast meals, 113 lunch meals, and 130 dinner meals. This 0.3% threshold was chosen after a sensitivity analysis (0.1–1.0%): headline outcomes (median RDI percent reduction, cluster structure) remained stable; see Section A.1.1 in S1 File. To address the sparsity presented in the dataset, i.e., many foods but only several foods per meal, we created a prototype mapping system using a nutrient-aware aggregation algorithm (Section A.1 in S1 File). This approach reduced the food space by 87.5% per subcategory we select prototypes via facility location so that weighted mass coverage ≥ 90% and nutrient fidelity (weighted cosine error or MARE) ≤ 7%, with assignment similarity floor 0.70 (full formulation in Section B in S1 File). Finally, we constructed bootstrap confidence intervals for the mean of each food in each meal-to-food data subset to reduce the list of ingredients to only those ingredients that were representative of the set of foods consumed. The lower bound of the confidence interval was used as a threshold for ingredient removal. This resulted in the filtered data subsets of 528, 627, and 686 foods to 39,435, 37,024, and 42,831 meals for breakfast, lunch, and dinner, respectively.

### Clustering analysis

We clustered meals separately by time-of-day (breakfast, lunch, dinner) to preserve meal-specific structure. Each meal was embedded in a hybrid, high-dimensional feature space that combined: (i) nutritional composition (macronutrients, fiber, energy, macro balance/density ratios) and (ii) categorical food composition (gram amounts per WWEIA main and subcategories, e.g., grains, fruits, vegetables, dairy, mixed dishes, snacks/sweets, beverages). Features were z-score standardized within meal type. We then applied an enhanced HDBSCAN [22,23] procedure with meal-type-specific parameters and post-processing cluster merging for stability and coverage. To profile clusters, we contrasted within-cluster vs. complement feature means and controlled multiple testing with Benjamini–Hochberg correction [24] at q ≤ 0.01. We required an absolute mean difference ≥ 0.15 for significance and labeled features “distinctive” at |Δ| ≥ 0.20, reporting *Cohen’s d* for effect sizes [25]. Detailed feature extraction, clustering parameters, statistical validation procedures, and cluster naming algorithms are described in Section A.2 in S1 File. This process produced stable, interpretable cluster archetypes for each meal type that were used as conditioning variables for generation and evaluation.

### Meal generation

Meal generation consisted of two stages: (1) We use a CVAE [26] with a 64-dimensional Gaussian latent z, and a FiLM-conditioned decoder with 3 Dense(512, GELU) blocks driven by concatenated cluster and meal-type embeddings (8 dimensions each). We include a learned pair-specific prior (log-odds of within-pair prevalence) and a hard allowed-foods gate (top-k by prevalence per pair, k = median item count; mask strength 12). Training uses Adam (lr = 5e-4, clipnorm = 0.5) with weighted BCE (dynamic pos-weight), KL (free-bits, annealed β with triangular cycling after warmup), and a count-matching term (full details and hyperparameters in Section B.1 in S1 File). And (2) a simple RDI‑per‑kcal portion assignment that converts presence probabilities to portions for USDA‑aligned reporting while preserving the model’s food combinations.

### Portion assignment

Meals are generated in gram units. Portions are initialized from canonical serving sizes (with small jitter) and refined by minimizing signed log₂ deviation from per-meal RDI-per-kcal targets with asymmetric penalties (higher weight for under-consumption of protein, fiber, and key micronutrients; higher weight for over-consumption of sodium, saturated fat, and sugars). Each meal type (breakfast, lunch, dinner) is assigned 25%, 35%, and 40% of a 2,000-kcal daily target, respectively (see Table H in S1 File). This allocation is a conventional three-meal distribution commonly used in dietary pattern and meal-occasion reporting (e.g., WWEIA); we assess robustness to alternative splits in Section B.4 in S1 File. The solution strategy is: (1) initialize portions from canonical serving sizes (with small jitter); (2) run iterative gradient steps to minimize the signed log₂ objective; (3) apply constraints in order: per-item minimum 5 g; group and per-item caps; energy retargeting to hit meal energy; upper-bound-only caps on sodium, sugars, and SFA; beverage grams and beverage energy fraction (≤25% kcal from beverages); soft total-grams cap (900 g) with rebalancing (Section B.2 in S1 File).

### Evaluation framework

We conducted a head-to-head evaluation of generated meals across 19,013 meals (6,268 breakfast, 6,393 lunch, and 6,352 dinner meals per significant clusters in Table F in S1 File), assessing established nutritional adequacy and diversity metrics [27–32]. Table A in S1 File shows the metrics used in greater detail. Preprocessing (LOF, nutrient-aware aggregation, and bootstrap-based ingredient filtering) and clustering were fit on the full development set; the CVAE was then evaluated under 5-fold stratified cross-validation on the resulting meal–cluster data. As a leakage-safe check, we re-estimated feature standardization and HDBSCAN clustering within each fold and report test-fold agreement with the baseline clustering in Section A.2.4 in S1 File. For analysis, *Cohen’s d* was used for effect sizes. Bootstrap resampling (1,000 iterations) generated confidence intervals including the headline RDI alignment (median percent reduction in deviation), cost savings and nutrition gains at substitution operating points, and nutrient-level summaries. FDR correction was applied for multiple comparisons. Model stability was assessed by 5-fold cross-validation.

### Meal substitution analysis

For each generated meal, we identify a small set of similar real meals to serve as candidate substitutions. Similarity is based on overall item overlap and composition, while requiring comparable meal energy and a similar number of items. We also consider simple single‑item replacements within the same food category. We define k as the number of items replaced. For each candidate, we compute nutritional improvement as the reduction in average absolute deviation from per‑meal Recommended Daily Intake (RDI) targets (25%/35%/40% of daily RDI for breakfast/lunch/dinner). Cost change is calculated using a portion-based restaurant-style pricing model: for each item we use a grams-per-portion and price-per-portion (from representative U.S. menu and industry portion benchmarks), apply per-item and cross-item caps (e.g., at most one soup bowl per meal), and add a fixed overhead of $2 per meal. Items not matching a named category use a generic side default (150 g, $3 per portion); apparent cost differences between dishes (e.g., some lamb-based vs chicken-and-rice) can therefore be portion- or category-driven rather than reflecting relative retail prices. Full specification and the implementation are given in Section C.1 in S1 File. An alternative grocery per-100g price map (S2 File) is available for sensitivity analyses. We select winning substitutions using a simple trade-off between improvements in nutritional adequacy and cost increases, with optional budget and “no cost increase” constraints. Substitution was attempted for all 19,013 generated meals in the evaluation set; 8,337 (43.8%) had at least one feasible candidate; the proportion with no feasible substitute (56.2%) and the full distribution of signed nutrition and cost changes (including the fraction of selected substitutes with higher cost) are reported in Section C.5 in S1 File. Portions in candidate real-meal matches are taken as observed; for single-item swaps, the grams of the removed item are reassigned to the added item.

## Results

### Meal clusters capture core U.S. meal archetypes across breakfast, lunch, and dinner

**Fig 2:**
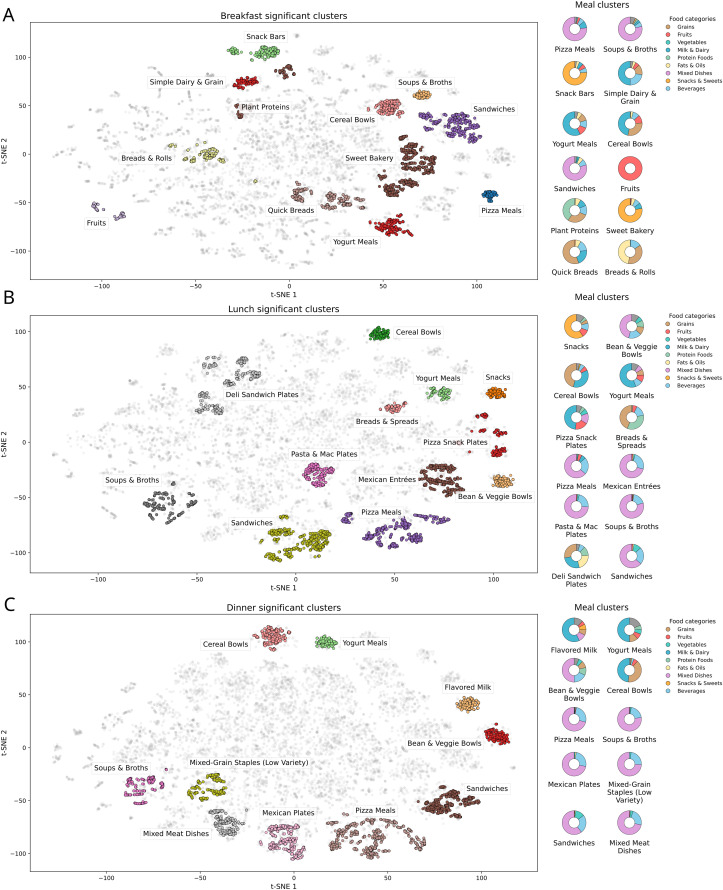
Meal archetypes across time-of-day in embedding space. t-SNE maps of meals from the hybrid feature space (nutrients and WWEIA category grams) show interpretable, compact clusters for breakfast (top), lunch (middle), and dinner (bottom). Grey points are background meals, and colored islands are clusters that pass false discovery rate (FDR)-controlled distinctiveness tests. Labels reflect the most distinctive food-group and nutrient signals (e.g., Cereal Bowls, Sandwiches, Pizza Meals, Soups & Broths). Donut charts at right summarize each cluster’s food-group composition (WWEIA main categories), illustrating the food-forward basis of the archetypes used for conditional generation and like-for-like evaluation.

We retained 34 interpretable clusters that map onto common U.S. meal archetypes (i.e., general meal patterns) and differ meaningfully in nutrition (Fig 2). Several clusters reveal behavioral trade-offs. For example a lunch breads & spreads pattern (Fig 2B) pairs breads with a very high fruit share (fruit ratio *Cohen’s d* = +12.41, q < 10 ⁻ ²⁷¹) and greater macro-diversity (*Cohen’s d* = +1.97, q < 10 ⁻ ¹¹⁴) but scores lower on overall meal balance (d = −2.05, q < 10 ⁻ ¹⁰³), suggesting that meals within have more snack-like plates rather than balanced entrée plates. Conversely, Mexican entrées concentrate on the main dish and trim the sides (ingredient count *Cohen’s d* = −0.74, q < 10 ⁻ ⁵²; portion variability d = −0.66, q < 10 ⁻ ³⁷), with strong mixed-dish enrichment (*Cohen’s d* = +4.47, q < 10 ⁻ ³⁰⁰). Together, these nutrition-anchored contrasts show that the retained clusters for training represent robust meal archetypes spanning energy-dense plates (pizza dinners, sandwich lunches, cereal breakfasts), leaner/fiber-positive options (yogurt across meal types, soups), and snack-style patterns (breads & spreads). Cluster sizes ranged from 210 to 1,871 meals per cluster (median 537), with 12 breakfast, 11 lunch, and 11 dinner clusters (Table F in S1 File). Subsample-and-recluster stability (Adjusted Rand Index vs. baseline) was high for breakfast (ARI median 0.83, 95% CI 0.81-0.84) and moderate for lunch (0.60, 95% CI 0.45-0.66); dinner clusters showed lower ARI (median 0.01; 0.05 when restricted to non-noise assignments), consistent with greater heterogeneity of dinner patterns (Section A.2.4 and Table L in S1 File).

### Generating realistic, uncompromised meals that improve RDI alignment

**Fig 3:**
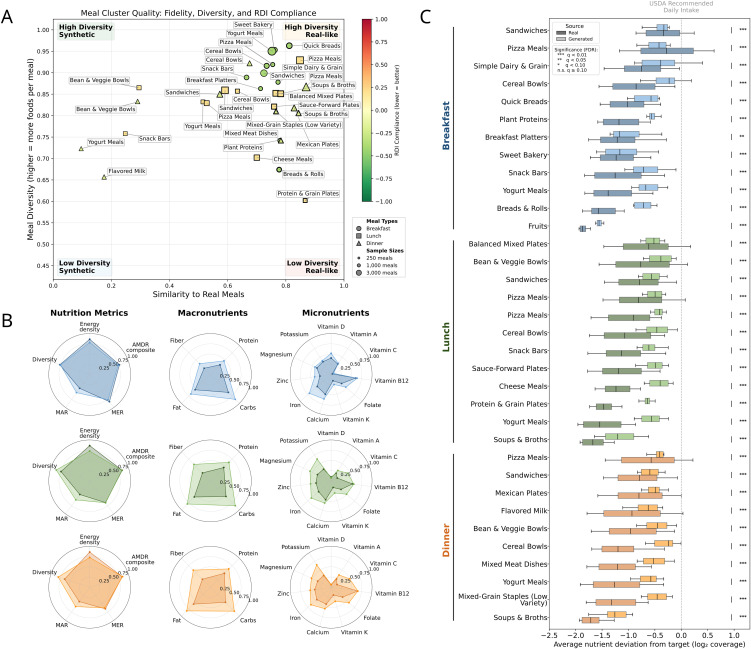
Generated meals improve nutritional targets while preserving variety. **A)** Similarity–item count map (axis: number of foods per meal; distinct from the Hill-index diversity in Table 1). Generated meals cluster within their archetypes yet remain widely distributed, indicating preserved variety and realism when matched by archetype. **(B)** Nutritional shifts by meal type. Relative to matched real meals, MAR (adequacy) increases, AMDR compliance (balance) improves, and energy density increases; selected micronutrients (e.g., vitamin C) and protein adequacy rise. **(C)** Deviation from USDA targets. Absolute per-meal median RDI deviation drops by 47.0% overall (43.2% breakfast; 52.1% lunch; 46.0% dinner), with significant improvements across most nutrients.

We compared generated and real meals within each meal‑archetype cluster using nonparametric bootstrap resampling (1,000 iterations per nutrient per cluster), counting an improvement when the 95% bootstrap interval for generated − real lay below zero. Generated meals concentrate in the high‑similarity region while preserving dispersion across clusters (Fig 3A), while nutrition-related characteristics are substantially improving (Fig 3B): adequacy increases across meal types (MAR: + 7.8% breakfast; + 51.3% lunch; + 14.4% dinner); median energy density is higher in generated meals (+104.1%, + 57.1%, + 31.9% for breakfast, lunch, dinner), reported descriptively (Table 1); and micronutrient coverage strengthens (e.g., vitamin C + 26.1%, + 136.2%, + 94.2%). Concomitantly, 97.1% of clusters show lower median deviation with respect to RDI recommendations for generated meals when compared to real same-cluster meals; the median percent reduction in cluster-level medians is 43.2% (95% CI: 11.2–51.6%) for breakfast, 52.1% (95% CI: 37.1–56.6%) for lunch, and 46.0% (95% CI: 32.9–60.9%) for dinner (overall median 47.0%; 95% CI: 11.2–60.9%; Fig 3C). An ablation that applies the same portion optimizer to real-meal food sets decomposes this gain into portion rebalancing and CVAE food-set selection (Table S in S1 File). At the nutrient level (Section B.5 in S1 File), fiber, protein, potassium, and most micronutrients show consistent improvements in median deviation from per-meal targets; by contrast, sodium shows increased deviation for generated meals at lunch and dinner. Total sugars and saturated fat are included in Section B.5 in S1 File, where real meals were already at or below target, percent reduction is not defined and we report deviation levels or mark as not applicable. The largest improvements to RDI score are in the Dinner Cereal Bowls (−80.1% reduction to the RDI deviation), Breakfast Cereal Bowls (−73.0%), and Lunch Deli Sandwich Plates (−67.6%); the only case where deviation was increased was in the Breakfast Pizza Meals (+50.8%). Thus, 33 of 34 clusters showed improved median RDI deviation for generated versus real meals, and the 47% median reduction was not driven by a small subset of archetypes.

**Table 1:** Primary evaluation metrics for generated meal assessment. Summary definitions and mathematical forms for the five principal metrics used to evaluate generated meals: Mean Excess Ratio (MER), Mean Adequacy Ratio (MAR), AMDR composite, dietary Diversity (Hill index; the “diversity” axis in Fig 3A is item count, not this metric), and Energy Density. In dietary guidance, lower energy density is often associated with more balanced diets; here we report energy density as a descriptive statistic, generated meals meeting per-meal energy targets may show higher values.

Metric	Definition	Formula	Notes
**Mean Excess Ratio (MER)**	Quantifies average proportional excess of overconsumed nutrients (e.g., sodium, saturated fat, added sugars) relative to upper dietary limits.	$\frac{\text{1}}{K}\sum_{k=\text{1}}^{K}\frac{I_{k}}{L_{k}}$	$I_{k}$: intake of nutrient k; $L_{k}$: recommended upper limit
**Mean Adequacy Ratio (MAR)**	Represents the average adequacy across essential micronutrients n, assessing overall nutrient sufficiency.	$\frac{\text{1}}{N}\sum_{n=\text{1}}^{N}NAR_{n}$	N=11 micronutrients (Ca, Fe, Zn, Vitamin A, C, B6, B12, Thiamin, Riboflavin, Niacin, Folate); $NAR_{n}=\text{min}(\text{1},\frac{I_{n}}{RDI_{n}})$
**AMDR Composite**	Evaluates macronutrient (protein, fat, carbohydrates) energy balance within acceptable macronutrient distribution ranges (AMDR).	$\frac{\text{1}}{M}\sum_{m=\text{1}}^{M}\text{1}(L_{m}\leq E_{m}\leq U_{m})$	$E_{m}$: percent of total energy from macronutrient m; $L_{m},U_{m}$: AMDR bounds (Protein 10–35%, Fat 20–35%, Carbohydrate 45–65%)
**Diversity**	Measures dietary variety across food groups F using the Hill index; higher values indicate broader food-group representation.	${\left(\sum_{i=\text{1}}^{F}\overset{q}{\underset{i}{p}}\right)}^{\frac{\text{1}}{\text{1}-q}}$	$p_{i}$: proportion of food group i; for q=1, $\text{exp}(-\sum_{i}p_{i}\text{ln}p_{i})$ (Shannon diversity)
**Energy Density**	Represents caloric content per gram of meal, reflecting portion appropriateness and caloric concentration.	$\frac{\text{Total Calories}}{\text{Total Mass }(\text{g})}$	Lower values correspond to less calorically dense and typically more balanced meals.

To evaluate the performance of our framework against state-of-the-art Large Language Models, we compared 3,400 meals (100 meals per 34 significant clusters) generated by our framework vs. similarly generated by GPT 4o (Section B.3 in S1 File). Results show that in all metrics except diversity, our framework over-performed GPT-4o, which is the most powerful LLMs at the time of the study, likely due to the high complexity and multi-objective nature of the task. More specifically, AMDR compliance was higher for the CVAE when compared to the LLM methods, with 18.9% vs 11.9%, respectively, of meals generated being AMDR compliant. Aggregating macronutrient composition across meals, our framework averaged 12.7% protein, 27.7% fat, and 61.6% carbohydrate, whereas GPT-4o averaged 16.8%, 39.4%, and 43.5%, and as such, GPT-4o’s meals were both high-fat (>35% fat content) and low-carb (<45%). Interestingly, GPT-4o produced more diverse meals (84%) than our framework (78%), which was not one of the objectives for this study. Overall, our framework better satisfies RDI/AMDR-based adequacy and macronutrient balance in the aggregate, while GPT-4o tends to emphasize compositional diversity.

### Food substitutions can make meals 10% healthier for 32% less cost

**Fig 4:**
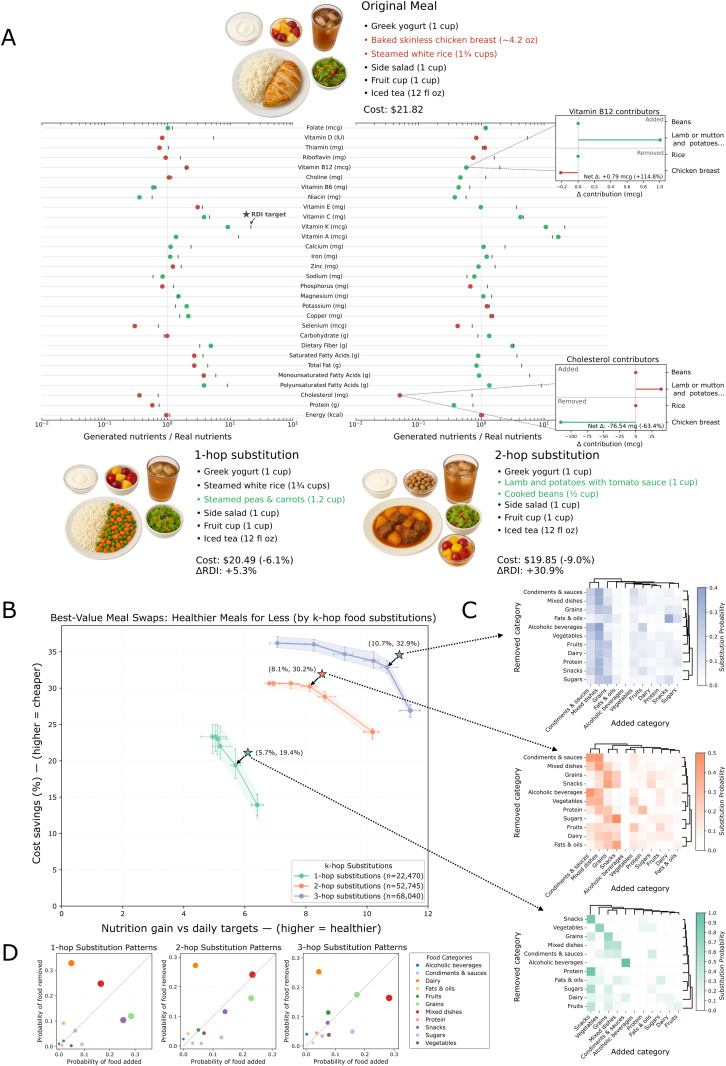
Cost–benefit of machine-guided meal substitutions. **(A)** A real meal (top) and two of its generated one-hop (bottom left) and two-hop (bottom right) substitutes. The middle plot shows per-nutrient ratios (substitute/original; log scale) with the vertical line at parity. Green points to the right indicate increased progress toward daily targets; red to the left indicate decreases. Insets show benefits of 2-hop swaps include a reduction in cholesterol and an increase in vitamins. **(B)** Population-level cost–benefit curves summarizing, for one-, two-, and three-hop substitutions, the trade-off between nutrition gain and cost savings as the policy parameter θ varies. Nutrition gain is the percent reduction in average absolute deviation from per-meal RDI targets (25%, 35%, and 40% of daily RDI for breakfast, lunch, and dinner, respectively). Stars indicate the selected operating points.; error bars or shaded regions show 95% bootstrap CIs (1,000 resamples) for median nutrition gain and cost savings. **(C)** Category transition heatmaps for each starred operating point. Each cell shows the probability that the row category is swapped for the column category. Warmer colors denote more frequent directed swaps. Marginal summaries indicate net adds/removes, and clustering exposes motifs (e.g., more additions from vegetables/grains and fewer removals from high cost/low value categories), consistent with the cost–benefit gains. **(D)** Category-level swap scatters at the same operating points. Each point is a category, with x-axis and y-axis denoting the probability for the added and removed category, respectively. Points on the diagonal reflect in-kind replacement; right/down shifts indicate net additions, up/left indicate net removals. With higher hop counts, patterns concentrate in add-heavy, easy-to-apply substitutions, consistent with an improved cost–benefit. Meal images shown in panel A were generated by the authors using ChatGPT/OpenAI and are not reproduced or adapted from third-party published or copyrighted sources.

Can we improve existing meals both nutritionally and cost-wise with few ingredient changes? To answer this question, we attempted substitution for all 19,013 generated meals in the evaluation set; 8,337 (43.8%) had at least one feasible candidate and are included in the cost–benefit analysis (3,000 one-hop, 4,146 two-hop, and 1,191 three-hop), drawn from 1,475 unique real foods. The proportion with no feasible substitute (56.2%) and the full distribution of signed nutrition and cost changes, including the fraction where the selected substitute increases cost, are reported in Section C.5 in S1 File. Fig 4A illustrates the per-meal accounting used throughout (nutrient difference and cost) for a specific meal with one and two substitutions (hops). At the knee of the cost–benefit frontier, substitutions deliver meaningful health improvements at lower cost across all hop settings. Nutrition gain is defined as the percent reduction in average absolute deviation from per-meal RDI targets (25%, 35%, and 40% of daily RDI for breakfast, lunch, and dinner). The representative knee points indicate nutrition gains of 5.2% (95% CI: 5.0–5.6%) with 22.0% (95% CI: 20.0–24.5%) savings for 1-hop, 8.1% (95% CI: 7.9–8.4%) with 30.2% (95% CI: 29.5–30.7%) savings for 2-hop, and 10.2% (95% CI: 9.9–10.4%) with 33.8% (95% CI: 32.9–34.9%) savings for 3-hop (Fig 4B). These points summarize the trade-off frontier; the full distribution of outcomes over all 8,337 selected substitutes is given in the Section C.5 in S1 File. Median cost savings and 95% bootstrap confidence intervals are reported in Section C.1 in S1 File and in the cost-sensitivity tables (Tables N and Table O in S1 File). The reported savings are relative to the stated portion-based pricing model; the pricing level is fixed at a single point in time and does not capture geographic or temporal variation. The composition of gains shifts systematically with hop count, with the number of hops correlating with the across-category by intra-category substitution per meal ratio, suggesting that the method must resort to solutions from other categories to maximize benefit when multiple substitutions are allowed (Fig 4C). With one-hop substitutions, improvements are nutrition-led, accompanied by moderate cost relief and small adjustments to items/portions. Allowing two-hop substitutions maintains strong nutrition contribution and raises ease of adoption, indicating many options that stay close to the original plate. Under three-hop, the center of mass pivots toward savings while nutrition’s share moderates; ease remains comparable. In other words, expanding flexibility widens access to deeper discounts while still improving nutrition, whereas tighter constraints emphasize nutrition-first improvements with smaller changes. Trade-off diagnostics are consistent with these patterns. Together, these results show that along the empirical frontier there are two practical operating modes, namely a local, nutrition-forward improvement with meaningful but moderate savings, to a more far-reaching, budget-forward improvements that achieve larger discounts while still moving nutrition upward.

## Discussion

In this work, we present a meal generation and substitution framework that aims to create practical, realistic meals that optimize nutrition and cost, while requiring only a few ingredient changes to existing meal choices. There are several methodological advances that facilitate its improved performance. First, segmenting the meal space into meal archetypes based on nutritional and co-occurrence embeddings has been key to allowing the focused training of the machine learning engine (the conditional variational autoencoder). Similarly, separating the meal generation from portion prediction improved nutritional alignment while preserving food diversity. Without these two architecture design changes, the F1-score from reconstructing the meal inputs in the autoencoder deteriorates from 0.99 to 0.64. To quantify meal quality, we constructed multi-objective metrics to evaluate both real and generated meals based on their nutritional alignment (RDI/AMDR), realism, and cost, and found that generated meals reduced the median deviation from per-meal RDI targets by 47.0% while remaining compositionally close to observed meals. Also, key to the whole design process was to start with combinations and servings that are currently and frequently consumed, rather than ab initio plate and portion sizes. For example, to predict portion sizes that are optimized for RDI, we optimize portions toward per-meal RDI targets (Section B.2 in S1 File) within realistic gram-level bounds, providing small, interpretable adjustments that correct nutrient macro- and micro-imbalances without distorting the underlying joint food combinations. Similarly, framing substitutions as a multi-objective trade-off, revealed operating points that can be tuned to user or program priorities – for example an interplay between health, affordability, or compromise to preferred choices. Head-to-head benchmarking against a LLM baseline further supports the value of embedding domain constraints directly into the generative process, and demonstrates higher standards-based performance (RDI/AMDR) than a free-form LLM approach.

Furthermore, since recommendations are derived from codified standards, the system could in future serve as one input to clinical decision support (e.g., during a dietitian-led visit or at discharge), with per-nutrient deviations and a concise “why this meal” rationale (archetype match, portion adjustments, and specific swaps) to inform, not replace, clinical judgment. Any such use would require integration with electronic health record (EHR) systems (e.g., via FHIR or HL7 interfaces) in a clinician-in-the-loop workflow, with the treating clinician retaining responsibility for final recommendations. Liability and safety considerations are critical: the tool is decision support only and must not be used to prescribe meals without addressing allergies, drug–nutrient interactions, and contraindications; clinical deployment would require validation and safety review. In addition, expanding this framework with a human-in-the-loop step would allow it to learn and adapt to preference signals and feedback, while keeping hard constraints, such as food allergies or clinical restrictions, in place. Similarly, guardrails can be included for populations at risk or with special nutritional needs (as for example, during pregnancy), and disease-specific recommendations, by adding extra terms and inequality constraints on the multi-objective optimization framework, and optimizing the weights for each such terms. Since one of the most important factors in whether a recommendation will be adopted both once and routinely is how difficult and different it is from the current practice, just-in-time adaptive interventions and a very well calibrated preference system that tunes in the difficulty grade of a substitution will be important. In this work we define substitution size operationally by the number of food items changed (k hops) and do not quantify real-world substitution burden (e.g., cooking method, retail availability, or cultural fit); an effort score or plausibility validation would strengthen behavior-change claims and is left for future work. Our current work performs the first step, by minimizing and keeping track of the number of hops; however, not all substitutions are equal, and integrating a “penalty” or “effort” matrix with both a general and personalized component is likely to increase the adherence to the proposed meals by minimizing the user burden.

The present evaluation is entirely computational (RDI alignment, cost model, diversity, and related metrics). We have not conducted human validation: usability of the system, interpretability of recommendations to users or clinicians, and “perceived burden or minimality” of substitutions have not been assessed with users or experts. The system cannot therefore be considered validated for clinical or consumer applications until such studies are conducted.

NHANES/WWEIA intake is self-reported and therefore subject to well-documented limitations, including measurement error (e.g., underreporting of energy and misreporting of portion sizes or items) and social desirability bias (e.g., overreporting of foods perceived as healthy and underreporting of snacks or sweets). These systematic biases can affect both archetype validity and model performance. Because archetypes are derived from reported nutritional composition and food-category structure, biased reporting can shift cluster boundaries, over- or under-represent certain meal patterns, and alter which meals are assigned to which archetype. The CVAE is trained on the same reported data, so generated meals and RDI-deviation metrics are relative to this reported baseline; improvements in RDI alignment (e.g., the 47% median reduction in deviation) do not correct for systematic under- or over-reporting in the source data. Interpretation of results should therefore treat archetypes and performance metrics as reflecting reported rather than true intake, and generalization to other populations or dietary assessment methods would require independent validation. Future work will extend this NHANES/WWEIA-based analysis, which relies on self-reported U.S. intake, to broader cultural settings and recipe-level granularity. The reported cost comparisons use a single point-in-time, U.S.-representative portion-based restaurant model (Section C.1 in S1 File); prices were set from representative menu and portion benchmarks and do not capture regional or temporal variability. While appropriate for population-level comparison of substitution strategies, a single, consistent pricing source would improve individual-level budgeting accuracy. Finally, we evaluate nutritional alignment and modeled costs rather than long-term adherence or clinical outcomes. Prospective, user-in-the-loop studies, potentially integrated with EHR/clinical decision support, should assess usability (e.g., task completion and satisfaction), interpretability of the recommendation rationale to users and clinicians, perceived burden or minimality of substitutions, adherence, Healthy Eating Index change, and condition-specific outcomes to establish real-world impact and readiness for deployment. These findings indicate a clear path toward actionable, guideline-consistent diet recommendations that respect preferences, budgets, and clinical constraints, while motivating prospective user-centered and clinical validation before real-world deployment.

## Supporting information

S1 File Supplementary materials and results, including Section A (Data analysis), Section B (Meal generation pipeline), Section C (Food substitution), Section D (Algorithmic implementation), and Tables A–S. (DOCX)

S2 File Grocery per-100g price map used for sensitivity analyses. (CSV)
