# Supplementary material for: Translating dietary standards into healthy meals with few-ingredient substitutions
Source: PLOS Digit Health. 2026 May 28;5(5):e0001367. doi: 10.1371/journal.pdig.0001367 (PMC13218539; doi:10.1371/journal.pdig.0001367)
Supplement: S1 File — (DOCX) [file pdig.0001367.s001.docx]

**Translating Dietary Standards into Healthy Meals with Few-Ingredient Substitutions**

Trevor Chan^1,2,3^ and Ilias Tagkopoulos^1,2,3*^

^1^Department of Computer Science, University of California, Davis, Davis, California 95616, USA

^2^Genome Center, University of California, Davis, Davis, California, 95616, USA

^3^USDA/NSF AI Institute for Next Generation Food Systems (AIFS), University of California, Davis, Davis, California, 95616 USA

*Corresponding author (itagkopoulos@ucdavis.edu)

Supplementary materials and results

A. DATA ANALYSIS

**A.1 Data Preprocessing**

We processed each meal type independently from ingestion through filtering, dimensionality reduction, and modeling. We reconciled, expanded, consolidated, and renumbered USDA food codes to their most recent identifiers using the published discontinuation mappings for 2013–2020^1^. Records from earlier years were excluded due to incomplete mappings. Dropped or revised codes were retained when their usage could be consistently interpreted across waves. This yielded a dataset with 120,375 meals and 6,212 foods across all meal types^2,3^. Within each meal-type, we applied Local Outlier Factorization^4^ with a neighborhood size appropriate for dense presence/gram matrices and a contamination rate of 0.3%. Scores were thresholded at the 99.7^th^ percentile to remove only the most extreme composition profiles. This step removed 120 breakfast, 113 lunch, and 130 dinner meals, respectively, while preserving central structure and variance. To mitigate sparsity and improve model tractability without sacrificing nutritional signal, we merged foods into prototypes using an aggregation method guided by nutrient profiles. The method used dynamic K allocation across WWEIA subcategories with meal-type-specific parameters (α=0.10, K_max=8 per subcategory, minimum subcategory size=6). Four criteria were applied to selection and assignment:

1. Mass coverage ≥ 90% of total consumed grams to ensure prototypes represent what people actually eat,
2. Nutrient fidelity with a ≤ 7% weighted mean absolute relative error across the nutrient panel used downstream;
3. Dynamic allocation of prototype counts across WWEIA subcategories (more capacity where heterogeneity is high),
4. Assignment quality with a cosine-similarity floor of 0.70 between a food and its prototype.

This procedure reduced the number of foods by 87.5% (6,212 foods to 777 foods) while maintaining macro- and micro-nutrient distributions at the meal type level. The nutrient-aware aggregation builds a prototype mapping (leaf food code to prototype code) as follows. (1) Per WWEIA subcategory, we run facility-location to choose up to K prototypes (K capped at 8 per subcategory; K allocated dynamically across subcategories). (2) Each leaf food is assigned to the prototype with highest cosine similarity in nutrient space; assignment is allowed only if similarity ≥ 0.70. (3) We choose the smallest K such that weighted mass coverage ≥ γ = 0.90 and weighted error ≤ ε = 0.07 (error = 1 − cosine similarity or, alternatively, weighted MARE in nutrient space). (4) Small subcategories (<6 foods) are rolled up to Main category and the same procedure is applied. The reported ~87.5% reduction is the ratio of distinct leaf foods to distinct prototypes in the resulting mapping. For each meal type, we computed the binary presence of each food across meals and constructed nonparametric confidence intervals for the mean food presence in meals using 1,000 bootstrap resamples with replacement. We retained foods whose empirical mean exceeded the lower 95% confidence bound and removed foods below that bound as non-representative. Meals that became all-zero after filtering were dropped. This yielded 39,435 breakfast meals with 528 foods, 37,024 lunch meals with 627 foods, and 42,831 dinner meals with 686 foods.

**A.1.1** **LOF contamination sensitivity.** The 0.3% LOF contamination threshold was justified by a sensitivity analysis. We varied the contamination rate over 0.1%, 0.3%, 0.5%, and 1.0% (evaluation-time sweep on the same preprocessed data and cluster labels). The median cluster-level percent reduction in RDI deviation (generated vs. real) remained stable across this range (see **Table Q**). We therefore chose 0.3% as a conservative default that removes only the most extreme composition outliers while retaining the vast majority of meals.

**A.2 Clustering Analysis**

**A.2.1 Feature Extraction.** For each meal type (breakfast, lunch, dinner), we constructed a hybrid feature set that includes (a) nutrient totals (protein, carbohydrate, total fat, fiber, energy), macronutrient percent-of-energy ratios and balance scores, log-transformed versions of skewed nutrients, and simple macro-interaction terms; and (b) gram amounts per WWEIA category and subcategory (15 main groups and 40+ subgroups; e.g., pizza, soups, cereals, savory snacks, sweetened beverages). We also included meal-level composition indicators (e.g., item count, portion variability, calorie density, food-category diversity). The complete feature set comprised 84 features per meal, including 5 core nutritional features, 16 derived nutritional features, 53 WWEIA category gram amounts (24 main categories and 29 subcategories), 5 enhanced composition metrics, and 5 transformed features (**Table E**) All features were standardized by z-scoring within meal type prior to clustering.

**A.2.2 Enhanced HDBSCAN Clustering.** We used HDBSCAN^5^ with a Euclidean metric and excess-of-mass (EOM) cluster selection method. Before clustering, we removed features with near-zero variance and those that were > 95% zero across meals to reduce noise. To favor generalizable clusters, we set meal-type-specific parameters: breakfast (min_cluster_size=50, min_samples=25, alpha=1.0, cluster_selection_epsilon=0.1), lunch (min_cluster_size=40, min_samples=20, alpha=1.0, cluster_selection_epsilon=0.08), and dinner (min_cluster_size=35, min_samples=18, alpha=1.0, cluster_selection_epsilon=0.06). These parameters were adjusted to reflect distributional differences (breakfast is typically more diverse while dinner is more homogeneous). After clustering, small clusters were merged into the most similar larger cluster using centroid similarity (merge when cosine similarity exceeded 0.7; otherwise assign to the nearest large cluster by Euclidean distance). This post-processing step increased coverage while preserving separability of dominant meal patterns.

**A.2.3 Statistical Validation.** For each cluster, we compared feature distributions against the complement set using a two-part hurdle approach: a prevalence test (non-zero vs. zero; Fisher's exact^6^ where applicable) and an intensity test on non-zeros (two-sided Mann–Whitney^7^). For each feature, we retained the p-value, then applied Benjamini–Hochberg FDR control at q ≤ 0.01^8^ across all cluster–feature pairs. We required a minimum absolute mean difference of 0.15 to declare significance and flagged distinctive features at |Δ| ≥ 0.20. We report Cohen's d^9^ to summarize effect sizes. Clusters dominated by beverages or exhibiting ultra-sparse compositions were deprioritized during interpretation to maintain food-forward, practically actionable archetypes. Cluster names were manually annotated by observing significant descriptors of each cluster. These clusters serve as conditioning variables in the generative model and as strata for evaluation.

**A.2.4 Cluster stability under resampling (ARI).** We assessed the stability of the meal archetype clustering by subsampling meals within each meal type, re-running HDBSCAN with the same parameters, and comparing the resulting partition to the baseline clustering using the Adjusted Rand Index (ARI). ARI ranges from 0 (no agreement) to 1 (perfect agreement). For each meal type we used 10 replicate subsamples (80% of meals for breakfast; 50% for lunch and dinner) and report the median ARI and 95% confidence interval across replicates. Breakfast clusters were highly stable (ARI median 0.830, 95% CI 0.812–0.841). Lunch clusters showed moderate stability (ARI median 0.597, 95% CI 0.455–0.661). Dinner clusters had low overall ARI (median 0.009, 95% CI 0.006–0.012) because a large fraction of meals fall in the “noise” (unassigned) label under HDBSCAN; when restricted to non-noise assignments only, dinner ARI was 0.055, indicating that assigned dinner clusters are somewhat consistent but that dinner patterns are more heterogeneous and sensitive to resampling than breakfast or lunch. These results support that the retained archetypes are stable for breakfast and lunch, and that dinner archetypes, while interpretable, are more variable under subsampling. To assess whether fitting clustering on the full dataset before CVAE cross-validation could materially overestimate performance, we performed a leakage-safe check using the same fold-based logic: for each fold we fit feature standardization (z-score) and HDBSCAN on training data only, then assigned held-out test meals to clusters via HDBSCAN’s approximate_predict and computed the Adjusted Rand Index between these test assignments and the baseline (full-data) clustering. Thus test data were never used to fit the cluster model. We report the median ARI and 95% CI across folds. Breakfast (3 folds): ARI(test) median 0.692 (95% CI 0.650–0.706). Lunch (2 folds): ARI(test) median 0.568 (95% CI 0.555–0.582). Dinner (2 folds): ARI(test) median 0.009 (95% CI 0.009–0.009); when restricted to non-noise assignments only, ARI(non-noise) median 0.055. These results indicate that when clustering is re-estimated on training data only, held-out test agreement with the baseline is moderate for breakfast and lunch and low for dinner (consistent with the subsampling analysis above), supporting that the baseline archetypes are not unduly driven by a single split. The CVAE is evaluated under 5-fold stratified cross-validation on the same baseline cluster labels; full nested preprocessing (LOF, aggregation, bootstrap filtering) within each fold was not performed and is left for future work.

**B. MEAL GENERATION PIPELINE**

**B.1 Food Composition Generation****.** We use a conditional VAE (CVAE)^10^ to predict the presence of foods, jointly conditioned on cluster and meal type. The encoder is an MLP (ReLU, 0.1 dropout) producing a 64‑dimensional Gaussian latent z. The decoder is a presence head with FiLM modulation^11^: 3 Dense(512, GELU^12^) blocks, each FiLM‑conditioned by the concatenated cluster and meal embeddings (8‑dim each). Two structural priors are installed: (i) a learned pair‑specific prior that boosts likely foods per (meal, cluster), and (ii) a hard allowed‑foods gate that zeroes disallowed items (also applied as a hard mask at inference). Training uses Adam^13^ (learning rate of 0.0005) and a batch size of 128 over 50 epochs. The loss is weighted binary cross‑entropy with dynamic positive‑class weighting plus KL with free‑bits^14^ and an annealed β schedule^15^ (linear warmup to 0.01, then light triangular cycling).

**B.2 Portion Assignment****.** For portion assignment, we employed an RDI-per-kilocalorie optimization. For each generated meal from the CVAE, foods with predicted presence probabilities above a threshold (p ≥ 0.02) are retained up to a maximum of 12 items. From the daily RDI vector $R$, we compute per-kcal nutrient densities

$$t_{k}=\frac{R_{k}}{R_{\mathrm{energy}}}$$

where $R_{\mathrm{energy}}=2,000\text{ kcal}$. Each meal $m$receives a fractional energy allocation $f_{meal type}\in\{breakfast: 0.25, lunch: 0.35, dinner: 0.40\}$, giving meal-scaled targets

$$r_{k}=t_{k}\text{ }f_{m}\text{ }R_{\mathrm{energy}}.$$

Vitamin D units are auto-converted (IU to µg) before normalization. We let $A\in\mathbb{R}^{n\times m}$ denote per-gram nutrient densities and $x\in\mathbb{R}^{n}$ the portions(g) of $n$ selected foods. The optimization minimizes asymmetric log₂ deviations from meal-scaled targets:

$$\min_{x\geq0}[\sum_{k} w_{k}(\log_{2}\frac{(Ax)_{k}}{r_{k}})_{+}^{\text{ }2}+\sum_{k^{'}} v_{k^{'}}(\text{ }\log_{2}\frac{r_{k^{'}}}{(Ax)_{k^{'}}})_{+}^{\text{ }2}],$$

where $w_{k}$ and $v_{k}$weight under- and over-consumption penalties (defaults listed in **Table J**). High-priority adequacy nutrients (energy, protein, fiber, potassium) receive greater weights (2.0), while moderation nutrients (sodium, added sugars, saturated fat) receive stronger penalties (3 to 4). Portions are constrained by:

- Total grams: ≤ 900 g per meal; soft downscaling if exceeded
- Beverages: ≤ 25% kcal of meal energy, with per-meal caps 300g/350g/350g (breakfast/lunch/dinner)
- Component caps: Sugars ≤ 12g, Fats/Oils ≤ 20g, Condiments/Sauces ≤ 20g, Snacks/Sweets ≤ 60g
- Per-item bounds: Solid foods ≤ 300 g each; minimum solid items = 2/3/3 (breakfast/lunch/dinner)

Optimization proceeds until the meal energy target is matched. Nutrient totals are re-projected if group caps bind. The final portion vector $x^{*}$defines a nutritionally coherent meal in grams. Derived nutrients are reported as $(x^{*}/100)^{\top}A_{100g}$.

**B.3 Comparison against state-of-the-art LLM.** We evaluated our CVAE against GPT-4o under matched conditions: same cluster-specific allowed foods, and the same nutritional and structural constraints used for CVAE portion assignment. Below we report the exact prompt protocol and post-processing so that the comparison is reproducible and fairness can be assessed. Both systems used the same WWEIA-derived nutrient matrix (per-gram nutrients) and food codes. For each (meal type, cluster) pair, GPT received exactly the set of food codes allowed for that pair in the CVAE (the "allowed foods" gate). No foods outside this set were permitted in GPT output; parsed responses that used other codes were discarded. An exact prompt that was used to generate breakfast meals is the following:


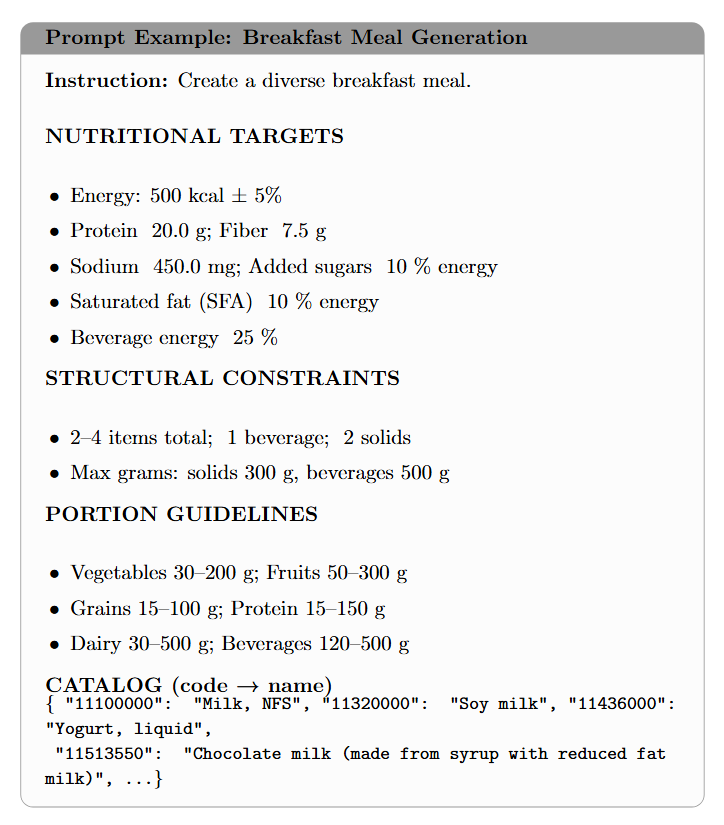


GPT responses were parsed as JSON. Only meals with all items having code in the allowed catalog and grams in (0, 1000] were retained; otherwise the meal was marked failed. No post-hoc portion optimization was applied to GPT output: gram amounts were used as returned by the model. CVAE meals were generated as presence probabilities then passed through the RDI-per-kcal portion optimizer (Section 2.2). We used batch generation (e.g., 50 meals per API call) with the same constraint template per (meal type, cluster). Default temperature and top_p settings for the API configuration were used.

**B.4** **Meal energy-split sensitivity (portion optimization).** To assess robustness of the main findings to the choice of meal energy allocation, we reran portion optimization under four energy splits (breakfast/lunch/dinner as % of daily 2,000 kcal): baseline 25/35/40, and alternates 30/35/35, 20/40/40, and 33/33/34 (approximate equal thirds). For each split and meal type we computed the median cluster-level percent reduction in median absolute deviation from per-meal RDI targets (Generated vs Real). Sample sizes were 1,200 meals (12 clusters) for breakfast and lunch and 1,000 meals (10 clusters) for dinner per split. (**Table K**). Across all splits, the median percent reduction in RDI deviation remained substantial (30–60%). The range by meal type across the four splits was: Breakfast 30.1% to 58.5%; Lunch 53.5% to 59.5%; Dinner 51.2% to 56.8%. Primary conclusions on improved RDI alignment of generated vs real meals are therefore robust to the choice of meal energy distribution.

**B.5 Nutrient-level outcome summary (Generated vs Real).** We report the percent reduction in median per-nutrient deviation from per-meal targets (positive indicates improvement; negative indicates worse). 95% CIs are bootstrap CIs over meals. Critical public-health moderation nutrients (sodium, total sugars, saturated fat) are included; for sodium, negative values indicate generated meals have higher deviation than real. For total sugars and saturated fat, when real meals were at or below target the percent-reduction metric is not applicable (**Table M**). Fiber, protein, and potassium show consistent improvements across meal types (roughly 10-67% reduction in median deviation), indicating that the portion optimizer and generated food combinations better meet adequacy targets for these nutrients. Sodium, by contrast, worsens at lunch and dinner (negative percent reduction): generated meals exhibit higher deviation from the per-meal sodium target than real meals, likely reflecting trade-offs when prioritizing energy balance, fiber, and other adequacy nutrients within the current constraints. For total sugars and saturated fat, real meals were often at or below the per-meal target (median deviation zero), so the percent-reduction metric is undefined; the optimizer does not systematically worsen these moderation nutrients where real meals were already compliant. Overall, the aggregate 47% improvement in RDI alignment is driven largely by gains in adequacy nutrients and macronutrient balance, while sodium remains a limitation for the current pipeline and may warrant stronger constraints or post-hoc caps in future work.

**B.6 Portion optimizer ablation (isolating CVAE vs. portion rebalancing).** The headline 47% median reduction in RDI deviation compares generated meals after RDI-per-kcal portion assignment to real meals as consumed. To separate the contribution of the generative model (CVAE food-set selection) from that of the portion optimizer, we applied the same RDI-per-kcal optimizer (Section 2.2) to real-meal food sets: for each real meal we kept the same foods and re-solved portions to minimize deviation from per-meal RDI targets under the same constraints (energy split 25/35/40%, gram caps, beverage limits, etc.). We then computed median absolute deviation from per-meal RDI for three conditions: (1) Real as-consumed, (2) Real and optimized (real food set, optimized portions), and (3) Generated and optimized (CVAE output with standard portion assignment). The median percent reduction in cluster-level median RDI deviation from Real as-consumed to Real and optimized (portion effect) was 6.9% (breakfast; 95% CI: -7.0% to 45.4%), 39.9% (lunch; 95% CI: 15.3% to 54.2%), and 26.7% (dinner; 95% CI: 10.6% to 49.6%). The incremental reduction from Real and optimizer to Generated and optimized (CVAE effect) was 13.9% (breakfast), 4.9% (lunch), and 12.0% (dinner), with an overall median across clusters of ~10%. The full-pipeline reduction (Real to Generated and optimized) was 38.9% (breakfast), 52.2% (lunch), and 46.0% (dinner), consistent with the 47% reported in the main text. This ablation confirms that the 47% headline improvement is not solely due to the portion optimizer; the CVAE's food-set selection contributes a meaningful incremental gain. Summary statistics are reported in **Table S**.

**C. FOOD SUBSTITUTION**

**C.1 Pricing Data.** We price meals using a portion-based restaurant model derived from public menu listings and industry guides. For each item we specify a grams-per-portion and a price-per-portion. Given the meal's actual grams, we compute a capped portion multiplier

$$m_{j}\text{ }=\text{ }\min\left( \frac{w_{j}}{g_{j}^{\left( \mathrm{portion} \right)}},\text{ }c_{j} \right),$$

The meal cost is the sum of (capped portions × price per portion) plus a configurable overhead

$$C\text{ }=\text{ }\sum_{j} m_{j}\text{ }p_{j}^{\left( \mathrm{portion} \right)}\text{ }+\text{ }\omega,$$

In the main analysis overhead was set to $2 per meal. Per-item and cross-item caps apply: e.g. at most one soup bowl and one fruit-salad serving per meal; items not matching a named category use a generic side default (150 g, $3.00 per portion). The named categories correspond to keyword-based rules on the food name (e.g. “soup”, “chicken breast”, “steamed white rice”) and were chosen to represent common restaurant-style components (soups, selected entrées and sides, bread, condiments, and beverages) and to keep the model tractable. All other foods are assigned the generic side default. As a result, apparent cost differences between meals can be portion- or category-driven rather than reflecting relative retail prices; this is a limitation when interpreting individual substitution examples. Selected portion and price pairs are given in **Table P.** Therefore, apparent cost differences between meals can be portion- or category-driven (e.g. a lamb-based dish priced as generic side versus chicken and rice with explicit entries) rather than reflecting relative retail prices per unit weight; this is a limitation when interpreting individual substitution examples. Portion sizes and prices are fixed per item category (no stochastic or regional variation). Prices reflect a U.S.-representative, point-in-time level (e.g. 2023–2024); no geographic or time-series variation is modelled. For the portion-based restaurant model (used for the main substitution cost results), grams-per-portion and price-per-portion are set from representative U.S. mid-scale and fast-casual menu listings and industry portion guides; specific values are implemented in `experiments/costs/restaurant_pricing.py`. The grocery per-100g matrix is used when running in grocery (per-100g) mode or for sensitivity; it includes source and justification columns (e.g. BLS CPI, representative U.S. grocers). We do not model geographic, seasonal, or store-level price variation. Cost estimates are intended for population-level comparison of substitution strategies. Confidence intervals for cost outcomes (median percent savings) are obtained by bootstrap over meals (1,000 resamples) and are reported in Section 3.4 and in the cost-sensitivity table below. Varying the overhead assumption ($0–$3) preserves the direction and approximate magnitude of cost savings (median savings 7.5-10.7% across overheads); see **Table N**. A grocery-only (per-100g) sensitivity, with no portion model and no overhead, is reported in **Table O**; median cost savings remain in the same direction and of comparable magnitude (e.g. 26–51% by hop count), supporting robustness to the choice of pricing structure.

**C.2 Evaluation**. We treat food substitutions in a meal as a constrained nearest-neighbor problem^16^ over real meals. From the real meal dataset, we analyzed 8,337 meals for which a generated substitute could be found (1-, 2-, or 3-hop), drawn from 1,475 unique real foods (Breakfast 3,224, Lunch 2,592, Dinner 2,521). For each generated meal, we form a candidate pool by retrieving the k-nearest real meals using a similarity that mixes presence Jaccard^17^ and portion-cosine^18^ (weight 0.7/0.3), under energy and item count. Similarity combines item overlap and portion composition; we require comparable energy (±5%) and similar item count (±1). For each candidate pair we compute edits and define $k_{\text{sub}}=\max\{\#\text{added},\text{ }\#\text{removed}\}$. Analyses are run separately at $k_{\text{sub}}\in\{1,2,3\}$ by enforcing exact $k_{\text{sub}}$at selection time. Subsequently, we define

- **Nutrition gain** $H_{i}$ as the positive change in percent of daily targets achieved. We use $H_{i}=\max\left( 0, \Delta RDI_{i} \right),$ so that negative changes do not contribute to “gain.”
- **Cost saving** $S_{i}$ as the positive percent reduction in price,

$$S_{i}=\max(0,\frac{Cost_{real}- Cost_{substitution}}{Cost_{real}}\times100\%).$$

- **Swap effort** $E_{i}\in[0,1]$ as the behavioral burden of the substitution, if taken. We combine (i) portion shift - the L_1_ change in ingredient weights, normalized by the meal’s total weight - and (ii) composition change - the larger of “added” or “removed” ingredients. Effort is $k_{\text{sub}}$‑aware - we normalize portion and composition changes against k‑based baselines, and set $E_{i}=\alpha\text{ }E_{portion}+\left( 1-\alpha\right)E_{composition}$, with $\alpha=0.5$. To note, this metric does not capture perceived burden, cooking method, retail availability, or cultural fit; incorporating those would require additional data and is left for future work.

**C.3 Winner Selection.** For a trade-off preference $\theta\geq0$, we map to a health weight $w=\frac{\theta}{1+\theta}\in[0,1]$. Each candidate receives a value

$$V_{i}(\theta)=wH_{i}\text{ }+\text{ }(1-w)S_{i}\text{ }-\text{ }w\text{CI}_{i},$$

where

- $H_{i}=\max(0,\Delta\text{RDI}_{i})$is the health gain in percentage points (reduction in mean absolute deviation from per-meal RDI targets).
- $S_{i}=\max(0,\frac{\text{Cost}_{\text{real}}-\text{Cost}_{\text{sub}}}{\text{Cost}_{\text{real}}}\times100\%)$ is cost saving (non-negative by construction).
- $\text{CI}_{i}=\max(0,\frac{\text{Cost}_{\text{sub}}-\text{Cost}_{\text{real}}}{\text{Cost}_{\text{real}}}\times100\%)$ is the cost-increase penalty (zero when the substitute is cheaper or equal).

We omit candidates that add or remove items from beverages or other main categories; pure portion-only edits are allowed but treated as within-category. Substitution winning candidates with the highest $V_{i}(\theta)$ are chosen in two stages:

1. Within-category stage. If any candidates keep the WWEIA main category matched between added and removed items (or only change portions), we shortlist them and pick the top by the value score below.
2. Cross-category challenge. A cross-category candidate may overtake the provisional within-category winner only if it is clearly better. If it adds any mixed dishes, it must exceed the within-category score by both a relative margin $\alpha=0.25$ and an absolute buffer $\beta=1.5$; otherwise a ≥ 20% relative uplift is required. If no within-category options exist, we select from the pooled set.

Ties are broken by (i) smaller portion-shift percentage, then (ii) larger health increase, then (iii) smaller cost savings. We ignore candidates with negative health change and drop negative overall scores when considering substitution candidates.

**C.4 Cost–benefit aggregation.** Sweeping $\theta$ across a grid spanning health-tilted to cost-tilted regimes, we select per-meal winners with $V_{i}(\theta)$, report the median nutrition gain $\tilde{H}(\theta)$ and median savings $\tilde{S}(\theta)$across meals, and form 95% bootstrap CIs over meals (1,000 resamples).

**C.5 Substitution outcome distributions.** To avoid optimistic reporting, we summarize signed substitution outcomes over all selected matches (not only frontier points). Substitution was attempted for 19,013 generated meals (same evaluation cohort as in the main text). Of these, 8,337 (43.8%) had at least one feasible candidate (1-, 2-, or 3-item substitution); the proportion with no feasible substitute is 56.2%. In the run reported, all selected substitutes had non-negative percent change in deviation from per-meal RDI targets. Overall by k: 1-hop n=3,000, median % Δ RDI deviation=+5.14, IQR=[2.20, 9.22]; 2-hop n=4,146, median % Δ RDI deviation=+7.56, IQR=[3.80, 13.52]; 3-hop n=1,191, median % Δ RDI deviation=+8.15, IQR=[4.37, 13.38]. Fraction of selected substitutes with cost increase: 28.8% (1-hop), 33.7% (2-hop), 29.6% (3-hop). Median percent change in cost: −16.95%, −17.92%, −21.49%. The fraction of meals with a selected substitute for which the chosen substitute has higher cost: overall by k 28.8% (1-hop), 33.7% (2-hop), 29.6% (3-hop); by meal type 25.1% (Breakfast), 34.1% (Lunch), 36.6% (Dinner).

**D. ALGORITHMIC IMPLEMENTATION**

Putting everything together, our framework, executes the following workflow.


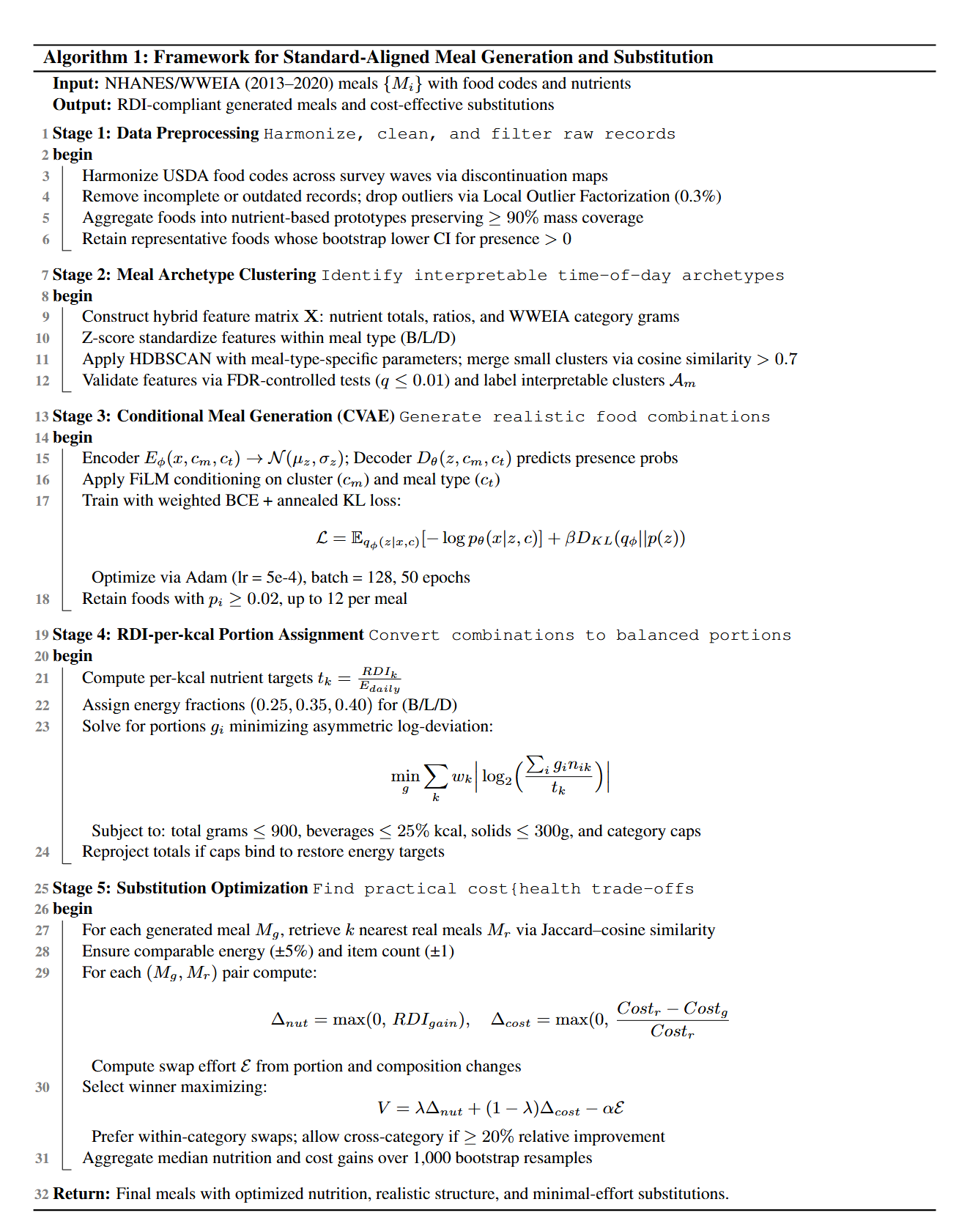


**REFERENCES**

1. 2019-2020 Food and Nutrient Database for Dietary Studies Documentation.

2. *NHANES Overview*. https://www.cdc.gov/nchs/nhanes/

3. *WWEIA (What We Eat in America)*. https://www.ars.usda.gov/northeast-area/beltsville-md-bhnrc/beltsville-human-nutrition-research-center/food-surveys-research-group/docs/wweia-documentation-and-data-sets/.

4. Breunig, M. M., Kriegel, H.-P., Ng, R. T. & Sander, J. LOF: identifying density-based local outliers. *SIGMOD Rec.* **29**, 93–104 (2000).

5. McInnes, L., Healy, J. & Astels, S. hdbscan: Hierarchical density based clustering. *JOSS* **2**, 205 (2017).

6. Fisher, R. A. On the Interpretation of χ^2^ from Contingency Tables, and the Calculation of P. *Journal of the Royal Statistical Society Series A (Statistics in Society)* **85**, 87–94 (2018).

7. Mann, H. B. & Whitney, D. R. On a Test of Whether one of Two Random Variables is Stochastically Larger than the Other. *Ann. Math. Statist.* **18**, 50–60 (1947).

8. Benjamini, Y. & Hochberg, Y. Controlling the False Discovery Rate: A Practical and Powerful Approach to Multiple Testing. *Journal of the Royal Statistical Society Series B: Statistical Methodology* **57**, 289–300 (1995).

9. Cohen, J. *Statistical Power Analysis for the Behavioral Sciences*. (Routledge, 2013). doi:10.4324/9780203771587.

10. Sohn, K., Lee, H. & Yan, X. Learning Structured Output Representation using Deep Conditional Generative Models. in *Advances in Neural Information Processing Systems* vol. 28 (Curran Associates, Inc., 2015).

11. Perez, E., Strub, F., Vries, H. de, Dumoulin, V. & Courville, A. FiLM: Visual Reasoning with a General Conditioning Layer. Preprint at https://doi.org/10.48550/arXiv.1709.07871 (2017).

12. Hendrycks, D. & Gimpel, K. Gaussian Error Linear Units (GELUs). Preprint at https://doi.org/10.48550/arXiv.1606.08415 (2023).

13. Kingma, D. P. & Ba, J. Adam: A Method for Stochastic Optimization. Preprint at https://doi.org/10.48550/arXiv.1412.6980 (2017).

14. Kingma, D. P. *et al.* Improving Variational Inference with Inverse Autoregressive Flow. Preprint at https://doi.org/10.48550/arXiv.1606.04934 (2017).

15. Chen, X. *et al.* Variational Lossy Autoencoder. Preprint at https://doi.org/10.48550/arXiv.1611.02731 (2017).

16. Cover, T. & Hart, P. Nearest neighbor pattern classification. *IEEE Trans. Inform. Theory* **13**, 21–27 (1967).

17. Jaccard, P. Étude comparative de la distribution florale dans une portion des Alpes et du Jura. https://doi.org/10.5169/SEALS-266450 (1901) doi:10.5169/SEALS-266450.

18. Manning, C. D., Raghavan, P. & Schütze, H. *Introduction to Information Retrieval*. (Cambridge University Press, 2008). doi:10.1017/CBO9780511809071.

**SUPPLEMENTARY TABLES**

|  | **Number of Records** | **Number of Respondents** | **Number of Meals** | | | | |
| --- | --- | --- | --- | --- | --- | --- | --- |
|  |  |  | **Breakfast** | | **Lunch** | | **Dinner** |
| NHANES 2013-2014 Day 1 | 131,394 | 8,661 | 21,331 | | | | |
|  |  |  |  |  |  |  |  |
|  |  |  | 7,019 | 6,594 | | 7,718 | |
| NHANES 2013-2014 Day 2 | 112,578 | 7,573 | 19,250 | | | | |
|  |  |  |  |  |  |  |  |
|  |  |  | 6,367 | 6,074 | | 6,809 | |
| NHANES 2015-2016 Day 1 | 121,481 | 8,505 | 20,501 | | | | |
|  |  |  |  |  |  |  |  |
|  |  |  | 6,789 | 6,298 | | 7,414 | |
| NHANES 2015-2016 Day 2 | 100,680 | 7,027 | 17,539 | | | | |
|  |  |  |  |  |  |  |  |
|  |  |  | 5,827 | 5,538 | | 6,174 | |
| NHANES 2017-2020 Day 1 | 183,910 | 12,632 | 30,242 | | | | |
|  |  |  |  |  |  |  |  |
|  |  |  | 9,914 | 9,210 | | 11,118 | |
| NHANES 2017-2020 Day 2 | 149,495 | 10,830 | 26,628 | | | | |
|  |  |  |  |  |  |  |  |
|  |  |  | 8,865 | 8,244 | | 9,519 | |
| **Total** | **799,538** | **55,228** | **135,491** | | | | |
|  |  |  |  |  |  |  |  |
|  |  |  | **44,781** | **41,958** | | **48,752** | |

**Table A |** Table showing the number of records, respondents, and meals per NHANES survey from 2013-2020.

| **Term** | **Definition** |
| --- | --- |
| Nutrients | the atomic units of meals e.g. vitamin C, Iron |
| Ingredients | composed of multiple nutrients, has a USDA ingredient code e.g. rice, seaweed |
| Food | composed of multiple ingredients, has a USDA food code e.g. sushi, sake |
| Meal | composed of multiple foods e.g. sushi with sake |
| Real Meal | meal that is consumed by real people (ground truth) |
| Practical Meal | meal that resembles a meal that is consumed by the population |
| Time-of-day | the eating occasion of a given meal e.g. breakfast, lunch, dinner |
| Healthy | as nutritionally close to USDA nutritional guidelines as possible |
| Recipe | ingredients with a list of instructions on how to cook them to become foods |

**Table B** | Definitions of meal composition framework

| **Discontinued Food Code** | **Description** |
| --- | --- |
| 1 – Dropped | Products no longer on the market; codes rarely used in the survey; items better coded using individual components as a combination. The specific products represented by some deleted codes have changed and no longer represent the food/beverage as described; however, similar products may be present within new/existing codes. |
| 2 – Expanded | New codes were created and the original code was discontinued. Modifications eliminated - products expanded to include presence/type of fat; codes expanded to designate source (restaurant, fast food, school); codes expanded by different ingredients. Codes representing more than one product or variety were expanded to individual codes. |
| 3 – Consolidated | Multiple codes now captured under a new/existing single code. The original codes were discontinued. |
| 4 – Renumbered | A food code was assigned a different 8-digit number yet represents the same product. Main food description may have been revised. |
| 5 – Revised | Codes received extensive revisions, including expansion and consolidation. |

**Table C |** FNDDS discontinued food codes.

|  | **Number of Meals** | | | | | | | **Number of Unique Foods** | | | | |
| --- | --- | --- | --- | --- | --- | --- | --- | --- | --- | --- | --- | --- |
|  | **Breakfast** | | | **Lunch** | | | **Dinner** | **Breakfast** | | **Lunch** | | **Dinner** |
| Raw Dataset | 135,491 | | | | | | | 7,679 | | | | |
|  | 44,781 | | 41,958 | | | 48,752 | | 4,346 | 6,063 | | 6,633 | |
| Food code standardization and filtration of unusable data | 134,457 | | | | | | | 6,599 | | | | |
|  | 44,440 | 41,628 | | | 48,389 | | | 3,881 | 5,361 | | 5,804 | |
| Outlier Filtration | 134,052 | | | | | | | 6,599 | | | | |
|  | 44,306 | 41,503 | | | 48,243 | | | 3,881 | 5,361 | | 5,804 | |
| Bootstrap Confidence Intervals | 134,052 | | | | | | | 6,591 | | | | |
|  | 44,306 | 41,503 | | | 48,243 | | | 3,872 | 5,356 | | 5,788 | |
| Food composition  Filtration | 65,202 | | | | | | | 2018 | | | | |
|  | 25,675 | 19,674 | | | 19,853 | | | 378 | 794 | | 846 | |

**Table D | The data visualization of the dataset size in terms of meals and their corresponding foods at each preprocessing step.** Table showing the preprocessing steps and resulting data dimensionality

| **Feature Category** | **Count** | **Reformatted Feature Names** | **Description** |
| --- | --- | --- | --- |
| **Core Nutritional Features** | 5 | Protein (g), Carbohydrate (g), Fat (g), Fiber (g), Energy (kcal) | Basic macronutrients and energy |
| **Derived Nutritional Features** | 16 | Protein Ratio, Carbohydrate Ratio, Fat Ratio; Protein Level, Carbohydrate Level, Fat Level, Energy Level; Protein–Carbohydrate Balance, Protein–Fat Balance, Carbohydrate–Fat Balance; Meal Balance Score, Nutritional Balance; Grain Ratio, Vegetable Ratio, Fruit Ratio, Dairy Ratio | Macronutrient and meal balance indicators |
| **WWEIA Main Categories** | 24 | Milk/Dairy, Mixed Dishes, Grains, Snacks/Sweets, Fruits, Vegetables, Beverages, Alcoholic Beverages, Water, Condiments/Sauces, Sugars, Baby Foods, Other; Milk, Flavored Milk, Dairy Drinks, Cooked Grains, Savory Snacks, Diet Beverages, Sweetened Beverages, Plain Water, Flavored Water, Baby Beverages, Human Milk | Main WWEIA categories |
| **WWEIA Subcategories** | 29 | Protein Foods, Fats/Oils, Cheese, Yogurt, Meats, Poultry, Seafood, Eggs, Cured Meats, Plant Proteins; Mixed Meat Dishes, Mixed Bean Dishes, Mixed Grain Dishes, Asian Dishes, Mexican Dishes, Pizza, Sandwiches, Soups; Breads/Rolls, Quick Breads, Cereals, Crackers, Snack Bars, Sweet Bakery, Candy, Desserts; Juice, Coffee/Tea, Infant Formulas | Protein, mixed dish, grain/snack, and beverage subcategories |
| **Composition Features** | 5 | Macronutrient Diversity, Food Category Diversity, Ingredient Count, Portion Variability, Calorie Density | Meal-level composition indicators |
| **Transformed Features** | 5 | log Protein (g), log Carbohydrate (g), log Fat (g), log Energy (kcal), log Fiber (g) | Log-transformed nutritional features |

**Table E | Summary of engineered meal-level feature categories used in the analysis.** This table lists all features derived from USDA and WWEIA mappings, grouped into six major categories. Core Nutritional Features capture basic macronutrient quantities and energy values. Derived Nutritional Features include macronutrient ratios, balance scores, and food group proportions. WWEIA Main and Subcategories represent hierarchical food groupings spanning milk, grain, fruit, vegetable, protein, and beverage domains. Composition Features quantify meal-level diversity, portion variability, and calorie density, while Transformed Features provide log-scaled nutrient representations for normalized statistical modeling.

| **Meal Type** | **Cluster Name** | **Number of Meals** | **Number of Foods** | **Top Categories** | **Max Absolute Cohen's d** |
| --- | --- | --- | --- | --- | --- |
| **Breakfast** | Pizza Meals | 238 | 59 | Pizza; Mixed dishes; Flavored milk; Sweetened beverages; Diet beverages | 43.32 |
|  | Soups & Broths | 223 | 59 | Soups; Mixed dishes; Plain water; Condiments sauces; Flavored milk | 14.19 |
|  | Snack Bars | 549 | 56 | Snack bars; Snacks sweets; Plain water; Yogurt; Mixed bean dishes | 15.57 |
|  | Simple Dairy & Grain | 277 | 49 | Other; Milk; Milk dairy; Plain water; Mixed bean dishes | 8.25 |
|  | Yogurt Meals | 590 | 51 | Yogurt; Milk dairy; Fruits; Plain water; Cereals | 9.77 |
|  | Cereal Bowls | 508 | 50 | Dairy drinks; Cereals; Milk dairy; Grains; Fruits | 8.23 |
|  | Sandwiches | 1122 | 55 | Sandwiches; Mixed dishes; Sweetened beverages; Beverages; Coffee tea | 8.66 |
|  | Fruits | 301 | 12 | Fruits; Mixed bean dishes; Flavored water; Savory snacks | 12.89 |
|  | Plant Proteins | 213 | 41 | Plant proteins; Breads rolls; Protein foods; Grains; Coffee tea | 4.36 |
|  | Sweet Bakery | 1871 | 45 | Sweet bakery; Snacks sweets; Coffee tea; Mixed bean dishes | 4.22 |
|  | Quick Breads | 711 | 38 | Quick breads; Grains; Sugars; Milk; Mixed bean dishes | 3.04 |
|  | Breads & Rolls | 396 | 35 | Breads rolls; Fats oils; Coffee tea; Sugars; Grains | 1.92 |
| **Lunch** | Snacks | 247 | 69 | Snack bars; Snacks sweets; Fruits; Crackers; Plain water | 16.53 |
|  | Bean & Veggie Bowls | 278 | 66 | Mixed bean dishes; Mixed dishes; Plain water; Meats; Cooked grains | 15.13 |
|  | Cereal Bowls | 271 | 67 | Cereals; Milk; Grains; Milk dairy; Sugars | 11.85 |
|  | Yogurt Meals | 218 | 61 | Yogurt; Milk dairy; Plain water; Cooked grains; Fruits | 8.85 |
|  | Pizza Snack Plates | 465 | 51 | Flavored milk; Milk dairy; Pizza; Poultry; Fruits | 5.6 |
|  | Breads & Spreads | 210 | 40 | Sugars; Plant proteins; Breads rolls; Grains; Protein foods | 4.28 |
|  | Pizza Meals | 1242 | 49 | Pizza; Mixed dishes; Sweetened beverages; Milk; Beverages | 5.46 |
|  | Mexican Entrées | 767 | 41 | Mexican dishes; Mixed dishes; Sweetened beverages; Plain water; Beverages | 4.26 |
|  | Pasta & Mac Plates | 527 | 38 | Mixed grain dishes; Mixed dishes; Plain water | 3.91 |
|  | Soups & Broths | 761 | 38 | Soups; Mixed dishes; Plain water | 4.34 |
| **Lunch**  **Dinner** | Deli Sandwich Plates | 703 | 44 | Cheese; Breads rolls; Cured meats; Grains; Milk dairy | 2.05 |
|  | Sandwiches | 1124 | 38 | Sandwiches; Mixed dishes; Sweetened beverages; Beverages; Condiments sauces | 4.02 |
|  | Flavored Milk | 352 | 70 | Flavored milk; Milk dairy; Sweet bakery; Pizza; Desserts | 24.54 |
|  | Yogurt Meals | 304 | 72 | Yogurt; Milk dairy; Cooked grains; Plant proteins; Plain water | 17.4 |
|  | Bean & Veggie Bowls | 369 | 64 | Mixed bean dishes; Mixed dishes; Plain water; Beverages; Mixed meat dishes | 10.8 |
|  | Cereal Bowls | 551 | 61 | Cereals; Milk; Milk dairy; Grains; Sugars | 9.54 |
|  | Sandwiches | 884 | 44 | Sandwiches; Mixed dishes; Sweetened beverages; Beverages; Condiments sauces | 4.93 |
|  | Pizza Meals | 1715 | 43 | Pizza; Mixed dishes; Sweetened beverages; Snack bars | 4.99 |
|  | Soups & Broths | 578 | 36 | Soups; Mixed dishes; Plain water; Snack bars | 3.82 |
|  | Mexican Plates | 850 | 37 | Mexican dishes; Mixed dishes; Sweetened beverages; Plain water; Snack bars | 3.72 |
|  | Mixed Meat Dishes | 537 | 38 | Mixed meat dishes; Mixed dishes; Plain water; Snack bars | 2.92 |
|  | Mixed-Grain Staples (Low Variety) | 504 | 31 | Mixed grain dishes; Mixed dishes; Plain water; Snack bars | 2.59 |

**Table F | Meal distributions per cluster.** The number of meals and unique foods per significant cluster, and the top contributing food categories. These meals form the basis for the training of the meal generation model (CVAE), and upon generation the same number of meals per cluster was used for evaluation.

| **Metric** | **Breakfast** | **Lunch** | **Dinner** |
| --- | --- | --- | --- |
| **F_1_ (micro)** | 0.9967 ± 0.0008 | 0.9969 ± 0.0007 | 0.9975 ± 0.0004 |
| **F_1_ (macro)** | 0.8337 ± 0.0396 | 0.8417 ± 0.0328 | 0.8486 ± 0.0342 |
| **Precision (micro)** | 0.9967 ± 0.0008 | 0.9969 ± 0.0007 | 0.9975 ± 0.0004 |
| **Precision (macro)** | 0.8393 ± 0.0441 | 0.8564 ± 0.0410 | 0.8877 ± 0.0149 |
| **Recall (micro)** | 0.9967 ± 0.0008 | 0.9969 ± 0.0007 | 0.9975 ± 0.0004 |
| **Recall (macro)** | 0.8291 ± 0.0397 | 0.8287 ± 0.0278 | 0.8189 ± 0.0473 |
| **AUROC** | 0.9844 ± 0.0022 | 0.9834 ± 0.0024 | 0.9740 ± 0.0023 |
| **AUPRC** | 0.7317 ± 0.0893 | 0.7447 ± 0.0764 | 0.7532 ± 0.0734 |
| **Brier score** | 0.00235 ± 0.00047 | 0.00223 ± 0.00040 | 0.00195 ± 0.00035 |
| **Count R²** | 0.7689 ± 0.0179 | 0.8562 ± 0.0289 | 0.7593 ± 0.0441 |
| **Count Bias** | 0.0763 ± 0.0077 | 0.0746 ± 0.0109 | 0.1198 ± 0.0204 |
| **True count (mean)** | 2.530 ± 0.014 | 2.480 ± 0.025 | 2.254 ± 0.016 |
| **Pred count (mean)** | 2.606 ± 0.007 | 2.555 ± 0.027 | 2.374 ± 0.029 |

**Table G | Performance of the presence-only CVAE model under five-fold cross-validation, reported as mean ± standard deviation across folds for each meal type**. Metrics include classification fidelity (F_1_, precision, recall), calibration (AUROC, AUPRC, Brier), and quantitative agreement in predicted food counts (R², Bias). Presence thresholds denote optimal probability cut-offs per fold, while “True” and “Pred count” indicate average numbers of foods per meal in ground-truth and model predictions, respectively.

| **Meal Type** | **Energy Fraction** | **Energy Target (kcal)** | **Description** |
| --- | --- | --- | --- |
| Breakfast | 0.25 | 500 | Represents 25% of daily 2000 kcal target |
| Lunch | 0.35 | 700 | Represents 35% of daily 2000 kcal target |
| Dinner | 0.4 | 800 | Represents 40% of daily 2000 kcal target |

**Table H | Meal energy-fraction assignments used for RDI-per-kcal portioning.** The fractional distribution of daily energy across breakfast, lunch, and dinner under a 2000 kcal pattern (0.25 / 0.35 / 0.40). These fractions are applied to both total caloric and per-kcal nutrient targets in the RDI-per-kcal optimization. The allocation is consistent with conventional proportional targets for three main meals used in dietary pattern and meal-occasion analyses; it ensures that each generated meal represents a defined share of daily intake.

| **Category / Subgroup** | **Cap (g)** | **Condition / Rule** | **Rationale** |
| --- | --- | --- | --- |
| **Total meal weight** | ≤ 900 | per meal | Limit total grams for realistic plate size |
| **Beverages (total)** | ≤ 25 % kcal or ≤ 300 / 350 / 350 g (B/L/D) | per meal | Avoid excessive beverage volumes |
| **Added sugars** | ≤ 12 g | absolute | Approx. 10 % kcal soft cap (per 2000 kcal diet) |
| **Fats / Oils** | ≤ 20 g | absolute | Prevent over-representation of oils/fats |
| **Condiments / Sauces** | ≤ 20 g | absolute | Maintain realistic side condiment levels |
| **Snacks / Sweets** | ≤ 60 g | absolute | Limit sweet or discretionary foods |
| **Solid food items (per item)** | ≤ 300 g | per item | Avoid unrealistic single-item servings |
| **Minimum # solid items** | 2 (B) / 3 (L) / 3 (D) | per meal | Ensure variety and realism |
| **Fluid dairy count as beverage** | – | inclusion rule | Included in beverage cap |

**Table I | Group- and per-item gram caps enforced during RDI-per-kcal optimization.** Gram-level caps and qualitative rules used to bound meal size, beverage volume, and individual component weights. These constraints prevent unrealistic portions while preserving dietary realism and nutrient feasibility. Beverage energy is limited to ≤ 25 % of meal kcal (300 / 350 / 350 g for breakfast, lunch and dinner), total meal mass is capped at ≤ 900 g, and per-item solids at ≤ 300 g. Additional limits on sugars, fats/oils, condiments, and snacks constrain discretionary energy. All caps are enforced softly via proportional rescaling when totals exceed limits.

| **Nutrient** | **Default RDI** | **RDI-per-kcal** | **Weight (Under) *wₖ*** | **Weight (Over)**  ***vₖ*** | **Constraint Type** |
| --- | --- | --- | --- | --- | --- |
| Energy (kcal) | 2,000 | 1 | 2 | 2 | Equality (fixed) |
| Protein (g) | 50 | 0.025 g/kcal | 2 | 1.5 | Adequacy |
| Carbohydrate (g) | 275 | 0.138 g/kcal | 1.5 | 1.5 | Adequacy |
| Total Fat (g) | 78 | 0.039 g/kcal | 1.5 | 1.5 | Adequacy |
| Fiber (g) | 28 | 0.014 g/kcal | 2 | 1 | Adequacy |
| Sodium (mg) | 2,300 | 1.15 mg/kcal | 1 | 3 | Upper bound |
| Saturated Fat (g) | 20 (10 % kcal) | 0.010 g/kcal | 1 | 3 | Upper bound |
| Added Sugars (g) | 50 (10 % kcal) | 0.025 g/kcal | 1 | 3 | Upper bound |
| Potassium (mg) | 4,700 | 2.35 mg/kcal | 2 | 1 | Adequacy |
| Calcium (mg) | 1,300 | 0.65 mg/kcal | 2 | 1 | Adequacy |
| Iron (mg) | 18 | 0.009 mg/kcal | 2 | 1 | Adequacy |
| Vitamin D (µg / IU) | 20 µg (800 IU) | 0.010 µg/kcal | 1.5 | 1 | Adequacy |
| Others (if present) |  |  | 1 | 1 | Neutral |

**Table J | Nutrient weights and per-kcal target values used in RDI-per-kcal optimization.** Nutrient-specific weights (wₖ, vₖ) and normalized RDI-per-kcal targets used in the generator’s objective function. Weights emphasize adequacy for key nutrients (energy, protein, fiber, potassium) and moderation for over-consumed ones (sodium, saturated fat, added sugars). RDI values correspond to FDA/USDA Daily Values for a 2,000 kcal reference diet. Per-kcal targets are scaled by meal-energy fractions before optimization. Together, these parameters guide the convex nutrient-anchored portioning that yields balanced, guideline-consistent meals.

| **Energy split (B/L/D %)** | **Breakfast** | **Lunch** | **Dinner** |
| --- | --- | --- | --- |
| 25 / 35 / 40 (baseline) | 46.8% | 55.1% | 56.8% |
| 30 / 35 / 35 | 53.4% | 56.6% | 52.4% |
| 20 / 40 / 40 | 30.1% | 59.5% | 55.7% |
| 33 / 33 / 34 (equal thirds) | 58.5% | 53.5% | 51.2% |

**Table K | Meal energy-split sensitivity**. Median percent reduction in RDI deviation by split and meal type. Median cluster-level percent reduction in median absolute deviation from per-meal RDI targets (Generated vs Real) when portion optimization is rerun under four energy splits (breakfast/lunch/dinner as % of daily 2,000 kcal). The baseline split is 25/35/40; alternates are 30/35/35, 20/40/40, and 33/33/34. Values show that improved RDI alignment of generated vs real meals holds across these splits.

| **Meal type** | **B** | **Subsample fraction** | **ARI median** | **ARI 95% CI (lower)** | **ARI 95% CI (upper)** | **ARI (non-noise) median** |
| --- | --- | --- | --- | --- | --- | --- |
| Breakfast | 10 | 0.80 | 0.830 | 0.812 | 0.841 | — |
| Lunch | 10 | 0.50 | 0.597 | 0.455 | 0.661 | — |
| Dinner | 10 | 0.50 | 0.009 | 0.006 | 0.012 | 0.055 |

**Table L | Cluster stability under resampling.**Adjusted Rand Index (ARI) comparing subsample-and-recluster partitions to the baseline clustering, by meal type. B is the number of replicate subsamples; the subsample fraction is the fraction of meals retained per replicate. For dinner, ARI(non-noise) restricts the comparison to meals assigned to a cluster (excludes HDBSCAN noise label). “—” indicates that the ARI was not computed for that meal type.

| **Nutrient** | **Breakfast** | **Lunch** | **Dinner** |
| --- | --- | --- | --- |
| Dietary fiber (g) | 39.6% [38.2, 41.4] | 60.9% [59.1, 62.3] | 64.2% [62.7, 66.2] |
| Potassium (mg) | 9.1% [5.9, 11.7] | 65.5% [64.0, 66.8] | 67.4% [66.2, 68.3] |
| Protein (g) | 35.5% [33.0, 37.3] | 51.4% [49.6, 53.0] | 46.9% [44.9, 48.8] |
| Sodium (mg) [moderation] | — | −80.3% [−88.5, −71.3] | −74.2% [−85.7, −65.4] |
| Total sugars (g) [moderation] | — | — | — |
| Saturated fat (g) [moderation] | — | — | — |

**Table M | Nutrient-level outcome summary (Generated vs Real).** Percent reduction in median per-nutrient deviation from per-meal RDI targets (25%/35%/40% by meal type), generated vs real meals. Positive values indicate improvement; negative values indicate higher deviation in generated meals. 95% CIs are bootstrap CIs over meals. ‘—' denotes not applicable (real meals at or below target).

| **Overhead ($/meal)** | ***n* meals** | **Median ΔCost (%)** | **Median savings (%)** | **95% CI (savings %)** |
| --- | --- | --- | --- | --- |
| 0 | 2,323 | -10.7 | 10.7 | 9.7 – 11.4 |
| 1 | 2,323 | -9.3 | 9.3 | 8.4 – 10.2 |
| 2 | 2,323 | -8.3 | 8.3 | 7.5 – 9.0 |
| 3 | 2,323 | -7.5 | 7.5 | 6.8 – 8.0 |

**Table N | Sensitivity of median cost savings to restaurant overhead (1-hop substitutions, portion-based pricing).** Median cost change (ΔCost) and median cost savings (percent reduction in meal cost) for 1-hop substitutions under the portion-based restaurant pricing model, by fixed overhead per meal ($0, $1, $2, $3). *n* is the number of meals with a selected substitution. 95% CIs for median savings are from bootstrap resampling over meals (1,000 resamples). Savings remain positive for all overhead values; magnitude decreases as overhead increases because the same dollar reduction is expressed relative to a larger baseline cost. Results are from the same substitution set with overhead applied for reporting only.

| **k (hops)** | **n meals** | **Median savings (%)** | **95% CI (savings %)** |
| --- | --- | --- | --- |
| 1 | 2,630 | 26.3 | 25.0 – 27.4 |
| 2 | 9,100 | 50.5 | 50.2 – 51.0 |
| 3 | 10,478 | 50.6 | 49.9 – 51.4 |

**Table O | Cost savings under grocery-only (per-100g) pricing by substitution hop count.** Median cost savings under grocery-only (per-100g) pricing (no portion-based model, no overhead), by number of substitution hops (k). Values are median percent cost reduction and 95% bootstrap confidence interval (1,000 resamples) over meals with at least one feasible substitute.

| **Category / item type** | **Grams per portion** | **Price per portion (USD)** |
| --- | --- | --- |
| Soup (bowl) | 350 | 6.00 |
| Fruit salad (cup) | 200 | 4.00 |
| Salisbury steak | 170 | 13.00 |
| Meatloaf | 170 | 12.50 |
| Chicken breast (baked) | 170 | 12.00 |
| Hot dog on bun | 120 | 4.00 |
| Whole wheat bread | 56 | 1.00 |
| Steamed white rice | 158 | 3.00 |
| Peas and carrots | 160 | 3.50 |
| Cooked beans | 170 | 4.00 |
| Shredded red cabbage | 70 | 3.00 |
| Cherries | 140 | 4.00 |
| Low-fat yogurt | 170 | 3.00 |
| Drinkable yogurt | 120 | 2.50 |
| Fruit juice | 240 | 2.50 |
| Ketchup | 17 | 0.10 |
| Mayonnaise | 14 | 0.20 |
| Iced coffee | 240 | 3.00 |
| Water | 240 | 0.00 |
| Generic side (default) | 150 | 3.00 |

**Table P | Portion and price pairs used in the portion-based restaurant pricing model.** Portion and price pairs used in the portion-based restaurant model. Item types are defined by keyword matching on the food name; foods not matching any named pattern use the generic side default (150g, $3.00).

| **Contamination** | **Breakfast** | **Lunch** | **Dinner** |
| --- | --- | --- | --- |
| 0.1% | 39.0% | 52.3% | 45.9% |
| 0.3% (chosen) | 39.0% | 52.7% | 45.8% |
| 0.5% | 39.0% | 53.1% | 45.8% |
| 1.0% | 39.1% | 53.1% | 45.9% |

**Table Q | Median percent reduction in RDI deviation (generated vs. real meals) by meal type and LOF contamination rate.** Sensitivity of the headline RDI-alignment metric to the LOF contamination parameter (evaluation-time sweep on the same preprocessed data and cluster labels). Each cell is the median across clusters of the within-cluster median absolute deviation from per-meal RDI targets for real meals vs. the same for generated meals, expressed as percent reduction. The chosen 0.3% rate removes only the most extreme composition outliers; results are stable across 0.1–1.0%.

| **Outcome** | **Point estimate** | **95% CI** |
| --- | --- | --- |
| **RDI alignment (median % reduction)** | | |
| Overall | 47.0% | [11.2–60.9]% |
| Breakfast | 43.2% | [11.2–51.6]% |
| Lunch | 52.1% | [37.1–56.6]% |
| Dinner | 46.0% | [32.9–60.9]% |
| **Substitution (knee points, θ=1)** | | |
| 1-hop: nutrition gain | 5.2% | [5.0–5.6]% |
| 1-hop: cost savings | 22.0% | [20.0–24.5]% |
| 2-hop: nutrition gain | 8.1% | [7.9–8.4]% |
| 2-hop: cost savings | 30.2% | [29.5–30.7]% |
| 3-hop: nutrition gain | 10.2% | [9.9–10.4]% |
| 3-hop: cost savings | 33.8% | [32.9–34.9]% |

**Table R | Primary outcomes with 95% bootstrap confidence intervals (1,000 resamples).** Point estimates and 95% CIs for (i) RDI alignment: median percent reduction in cluster-level median absolute deviation from per-meal RDI targets (generated vs. real meals), overall and by meal type (breakfast, lunch, dinner); (ii) substitution analysis: at the θ = 1 operating point, median nutrition gain (percent reduction in mean absolute per-meal RDI deviation) and median cost savings (percent), by hop count (1-, 2-, or 3-hop). RDI CIs from the portion-optimizer ablation (generated and optimized); substitution CIs from nonparametric bootstrap over meals in the substitution match files. All CIs are two-sided 95% intervals.

| **Condition** | **Breakfast** | **Lunch** | **Dinner** | **Overall (median)** |
| --- | --- | --- | --- | --- |
| Real and optimized (portion effect) | 6.9%  (−7.0 to 45.4) | 39.9%  (15.3 to 54.2) | 26.7%  (10.6 to 49.6) | ~18% |
| Generated and optimized (full pipeline) | 38.9%  (11.2 to 51.6) | 52.2%  (37.1 to 56.6) | 46.0%  (32.9 to 60.9) | ~47% |
| Incremental CVAE (Real and optimized to Generated and optimized) | 13.9% | 4.9% | 12.0% | ~10% |

**Table S | Portion optimizer ablation.** Portion optimizer ablation. Median percent reduction in cluster-level median absolute deviation from per-meal RDI targets by condition and meal type (95% bootstrap CI in parentheses where applicable). Overall is the median across the 34 clusters.
